# Effectiveness of Deep Brain Stimulation for Treatment-Resistant OCD: Systematic Review of Anatomical Targets and Meta-Analysis of Randomized and Non-Randomized Studies

**DOI:** 10.3390/brainsci16080789

**Published:** 2026-07-27

**Authors:** Sébastien Dufault, Stéphane Potvin, Simon Patry

**Affiliations:** 1 Department of Psychiatry and Addictology, University of Montréal, Montreal, QC H3T 1J4, Canada; sebastien.dufault@umontreal.ca (S.D.); stephane.potvin@umontreal.ca (S.P.); 2 Centre de Recherche, Institut Universitaire en Santé Mentale de Montréal, Montreal, QC H1N 3V2, Canada; 3 Department of Psychiatry, Institut Universitaire de Santé Mentale de Qubec, Quebec, QC G1J 2G3, Canada

**Keywords:** deep brain stimulation, DBS, obsessive compulsive disorder, OCD, DBS-targets, anxiety, depression

## Abstract

**Highlights:**

**What are the main findings?**

- DBS significantly reduces OCD symptom severity across both randomized controlled trials and non-randomized studies, with large concomitant improvements in comorbid anxiety, depression, and global functioning in severe treatment-resistant OCD.
- Therapeutic effects persisted beyond 12 months, and follow-up duration was the sole significant meta-regression predictor. Effectiveness varied more than twofold across stimulation targets, underscoring the clinical relevance of target selection.

**What are the implications of the main findings?**

- Symptom reduction in TR-OCD was sustained for at least 30 months, with improvements in comorbid anxiety, depression, and global functioning. These findings suggest that short follow-up durations in clinical trials may underestimate the full therapeutic potential of DBS.
- The longstanding practice of defining DBS targets by intended anatomical label rather than verified active contact location may have obscured clinically meaningful differences in therapeutic effectiveness. Standardized anatomical reporting is a prerequisite for optimizing surgical decision-making and designing definitive randomized trials.

**Abstract:**

Background: Deep brain stimulation (DBS) is a promising intervention for severe treatment-resistant OCD (TR-OCD), yet evidence of clinical efficacy remains inconsistent across heterogeneous study designs and anatomical target selection lacks standardization. Whether target choice influences clinical outcomes has not been systematically established, and prior meta-analyses have not characterized target-specific effects or predictors of response. Objectives: This systematic review and meta-analysis evaluated the efficacy of DBS versus sham stimulation in RCTs and its effectiveness in non-randomized studies, with OCD symptom severity, comorbid anxiety and depression, and global functioning as outcomes. Secondary objectives were to examine whether anatomical stimulation site influences therapeutic outcomes using systematic recategorization of reported targets, and to characterize the temporal dynamics of treatment response. Methods: Four databases (PubMed, Embase, Web of Science, CENTRAL) were searched through July 2025 following PRISMA 2020 guidelines. RCTs (active vs. sham DBS) and non-randomized studies reporting pre/post-DBS Y-BOCS scores were analyzed separately at three time points (≤12 months, >12 months, last follow-up). Stimulation targets were recategorized using active contact location and stereotactic coordinates. Random-effects models, meta-regression, RoB 2, ROBINS-I, and GRADE were applied. Results: Eight RCTs (*n* = 83) and 32 non-randomized studies (*n* = 321) were included. In RCTs, active DBS reduced Y-BOCS scores by 6.92 points (95% CI: 4.46–9.38; 18.3%; *p* < 0.001; I^2^ = 62.93%) versus sham. Non-randomized studies demonstrated reductions of 13.83 points (95% CI: 11.01–16.52), 17.34 points (95% CI: 14.70–20.04), and 14.51 points (95% CI: 12.78–16.26; 43.5%; *p* < 0.001) at ≤12 months, >12 months, and last follow-up, respectively, with substantial heterogeneity (I^2^ ≥ 89.79%). Responder and remission rates were 60.2% (95% CI: 52.8–67.2%; I^2^ = 20.7%) and 36.4% (95% CI: 29.8–43.5%; I^2^ = 12.1%), respectively. Anxiety (*k* = 7; *d* = 1.1; 95% CI: 0.72–1.56), depression (*k* = 24; *d* = 1.1; 95% CI: 0.80–1.26), and global functioning (*k* = 14; baseline GAF = 34.5; MD = 25.62; 95% CI: 22.18–29.03) improved significantly (all *p* < 0.001). In meta-regression, longer follow-up duration was the only significant predictor of Y-BOCS improvement (*p* = 0.042). Therapeutic effects varied substantially across anatomical stimulation sites. The anterior limb of the internal capsule (ALIC) and its ventral subdivision (vALIC) were most frequently stimulated. The inferior thalamic peduncle was associated with the largest mean Y-BOCS improvement (19.70 points; based on two trial arms only), nucleus accumbens stimulation did not reach the clinical response threshold (≥35% Y-BOCS reduction), and mediodorsal/ventral anterior thalamic stimulation showed no statistically significant benefit. Sensitivity analyses confirmed the robustness of primary estimates. Evidence quality was moderate (RCTs) and low (non-randomized studies). Conclusions: DBS significantly reduces OCD symptom severity in TR-OCD despite substantial heterogeneity, with greater improvements associated with longer follow-up duration and effects maintained for at least 30 months, alongside improvements in comorbid anxiety, depression, and global functioning. Not all anatomical stimulation sites are therapeutically equivalent, underscoring the importance of target selection. High-quality RCTs with standardized anatomical reporting are needed to consolidate the evidence base.

## 1. Introduction

Obsessive compulsive disorder (OCD) is a complex neuropsychiatric condition characterized by recurrent obsessions, compulsions, or both [1,2,3]. Obsessions are intrusive, distressing thoughts, whereas compulsions are repetitive or ritualistic behaviors performed to alleviate anxiety. Symptom dimensions in OCD are heterogeneous and vary considerably across individuals [4]. In most cases, OCD begins during childhood or adolescence and tends to persist into adulthood [5]. Recent data estimate the global lifetime prevalence of OCD at 4.1%, with higher rates in low- and middle-income countries (4.9%) and lower rates in high-income countries (3.4%) [5], establishing OCD as one of the most prevalent neuropsychiatric conditions worldwide [6]. Associations have been reported between childhood emotional trauma and illness severity [7]. OCD often leads to substantial impairments in social and occupational functioning and is frequently comorbid with anxiety disorders and major depressive disorder (MDD), with elevated rates of suicidal ideation compounding overall disability [8].

The pathophysiological mechanisms of OCD remain unclear, with several theories emphasizing its multidimensional nature [2]. OCD is increasingly recognized as a network disorder [9], involving reward, affective, cognitive-control, and default mode networks [10,11]. Evidence indicates structural and functional alterations, neurophysiological abnormalities [12], and changes in neuronal connectivity [13], potentially contributing to disrupted functional connectivity. Dysregulation between excitatory direct and inhibitory indirect pathways leads to affective network overactivation and reward-seeking network impairment, causing hyperactivity of the cortico-striato-thalamo-cortical (CSTC) circuit [10,11,14].

First-line treatments for OCD include a combination of selective serotonin reuptake inhibitors or tricyclic antidepressants, along with cognitive-behavioral therapy [15]. However, 10–30% of patients remain refractory to treatment [15]. For treatment-resistant OCD (TR-OCD), ablative surgical techniques, such as capsulotomy, anterior cingulotomy, and radiofrequency lesioning, have been practiced since the 1950s [16,17], aiming to disrupt abnormal connectivity in the frontothalamic fibers of the anterior limb of the internal capsule (ALIC) [16,17,18]. Approximately half of patients treated with ablative procedures showed a ≥35% reduction in symptom severity at one year [17,18,19]. Thermocapsulotomy yields response rates of approximately 47% [20] and mean Y-BOCS improvements of 31–81% across series [17,21], while Gamma Knife anterior capsulotomy produces improvements in 55–70% of patients [22]. Permanent or serious adverse events, however, occur in up to 21.4% of cases following capsulotomy [22,23].

DBS has emerged as a promising treatment for severe TR-OCD, offering a valid alternative to ablative procedures with comparable outcomes [19,24]. This adjustable, partially reversible technique, with a well-established safety and efficacy profile in movement disorders [25], may modulate the dysfunctional CSTC circuit by inhibiting pathological activity across different spatiotemporal levels [26]. In TR-OCD specifically, DBS has demonstrated an acceptable safety profile, as characterized in prior systematic reviews [27,28,29,30]. Hardware-related complications occur in approximately 8% of cases, encompassing lead migration, lead damage, and device malfunction; postoperative infection and seizure are reported in 4.4% and 3.6% of patients, respectively [29]. Stimulation-induced psychiatric adverse events, including transient hypomania and increased anxiety, are among the most frequently reported, typically resolving spontaneously or following stimulation parameter adjustments, with rare cases requiring hospitalization or modification of the stimulation target [27,28,29,30]. Suicide attempts have been reported (2.4%) [29]; one fatality due to suicide has also been documented, though direct attribution to DBS remains uncertain given the elevated baseline risk of suicidality in severe TR-OCD [28,29,30]. Serious adverse events, including intracranial hemorrhage, are rare (1.6%) [29].

Nuttin et al. (1999) [31] were among the first to report the benefits of DBS for OCD targeting the ALIC. Since then, several anatomical structures have been investigated, including the ventral striatum (VS), nucleus accumbens (NAc), bed nucleus of the stria terminalis (BNST), antero-medial subthalamic nucleus (amSTN), inferior thalamic peduncle (ITP), and medial-dorsal/ventral-anterior (MD/VA) thalamic nuclei. Neuroimaging studies using tractography and connectomics suggest that modulating projection pathways within white matter tracts, such as the superolateral branch of the medial forebrain bundle and ventral tegmental area (slMFB/ventral tegmental area) or the anterior thalamic radiation (ATR), may be more effective than targeting a single focal structure [32,33,34,35]. Whether distinct stimulation targets differentially modulate specific OCD symptom dimensions remains an open question with potential implications for patient selection.

Coenen et al. [10] identified eight subcortical projection pathways and categorized them according to their predominant involvement in large-scale brain networks. The slMFB/ventral tegmental area projection pathway is associated with the reward network, while ATR projection pathway is associated with the affective network. Both converge in the vALIC to project to the orbitofrontal cortex. The STN, substantia nigra, and red nucleus projection pathways are associated with the cognitive control network and converge in the dorsal half of the ALIC to project to the dorsolateral prefrontal cortex.

Some studies initially reported DBS targets based on the intended structure, such as the NAc, rather than on the anatomical locations of the active electrode contacts [36,37]. Given the anatomical configuration and the overlap between structures, it is common for the electrode trajectory to traverse multiple regions, particularly along the striatal axis [38]. Depending on the volume of tissue activated and the configuration of active contacts, stimulation may concurrently extend to adjacent structures and interconnected networks to varying degrees (e.g., VC/VS region, NAc as the ventral extension of VC/VS, and ALIC) [34,39,40].

Response to DBS for TR-OCD varies across studies, with outcomes potentially influenced by the stimulation target, parameters, study methodology, trial and follow-up duration, and patient-specific factors (comorbidities and phenotypes). While prior meta-analyses have addressed DBS effectiveness for TR-OCD, including effects on comorbid depressive symptoms [27,29,41] and anxiety symptoms and global functioning [41], important questions remain. Since the last meta-analysis [29,41], at least six new retrospective studies have been published [35,42,43,44,45,46].

This systematic review and meta-analysis evaluated the efficacy of DBS versus sham stimulation in randomized controlled trials, and its effectiveness in non-randomized studies at short-term (≤12 months), long-term (>12 months), and last follow-up time points, using the Y-BOCS as the primary outcome. Response and remission rates were assessed as secondary outcomes. It also examined the effects of DBS on comorbid anxiety and depressive symptoms, as well as global functioning. Additionally, the therapeutic effectiveness across stimulation targets was examined based on stereotactic coordinates and anatomical localization of active contacts, as reported by the study authors, rather than relying solely on the intended target structure. Inconsistent anatomical nomenclature across studies was systematically addressed [40]. Finally, risk of bias was assessed separately for RCTs and non-randomized studies, and the overall certainty of evidence was evaluated using the Grading of Recommendations, Assessment, Development and Evaluation (GRADE) [47] approach.

## 2. Materials and Methods

This meta-analysis adhered to the 2020 Preferred Reporting Items for Systematic Reviews and Meta-Analyses (PRISMA) guidelines, using the PRISMA checklist [48].

### 2.1. Search Strategy and Selection Criteria

A systematic search of PubMed, Web of Science, Embase, and the Cochrane Central Register of Controlled Trials (CENTRAL) was conducted by two reviewers from inception to 20 July 2025, without date restrictions. Search terms included controlled vocabulary and free-text terms for OCD (e.g., Obsessive Compulsive Disorder, OCD, TR-OCD, obsessive compulsive neurosis) and DBS (e.g., Deep Brain Stimulation, DBS, brain stimulation, neurostimulation). Clinical trial registries were not searched. Search queries are detailed in Supplementary Tables S1 and S2. Additional studies were identified through manual reference list searches and cross-referencing with prior systematic reviews and meta-analyses [27,29,41].

We included RCTs comparing DBS-ON with sham (DBS-OFF) and non-randomized studies (open-label extension phases, cohort studies, observational studies, and case series) that met the following criteria: (1) adult participants (≥18 years) with a primary diagnosis of OCD diagnosed per DSM-IV/V or ICD; (2) DBS as the primary intervention; (3) stimulation target reported; (4) OCD symptom severity reported as a primary outcome; (5) Y-BOCS scores reported at pre- and post-treatment time points; (6) English-language, peer-reviewed publication. Studies were included regardless of comorbidities or year of publication.

We excluded (1) reviews, meta-analyses, letters, editorials, and technical reports; (2) single-patient case reports; (3) DBS studies for indications other than OCD; (4) studies with neurophysiological, electrophysiological, neuropsychological, or behavioral primary outcomes only; and (5) animal studies.

### 2.2. Selection Process and Data Collection

Duplicate records identified across all four databases were removed using EndNote 21 and Zotero (version 9.0.6), and the remaining records were exported to a standardized data extraction form. Titles and abstracts were screened independently by two reviewers, and full texts of potentially eligible records were then retrieved and assessed for eligibility (*n* = 147). Disagreements were resolved by consensus.

Overlapping cohorts were identified by cross-checking study characteristics, recruitment periods, study sites, and participant demographics. Where multiple publications reported data from the same patient cohort, a single dataset was retained. For non-randomized studies, the publication reporting the longest follow-up with the most comprehensive outcome data was prioritized.

Three units of analysis were distinguished throughout: study (a unique publication), trial arm (a distinct stimulation condition or anatomical target contributing an independent effect estimate), and participant (a unique enrolled individual). RCTs and non-randomized studies were analyzed as distinct analytical strata, with participant counts maintained independently within each. In crossover RCTs, only participants completing both active (DBS-ON) and sham (DBS-OFF) conditions with available Y-BOCS data were retained. For studies spanning both a randomized and an open-label phase, participants completing the randomized phase contributed to the RCT analysis, and those with available open-label data contributed to the LFU analysis. In Luyten et al. (2016) [49], for example, 17 of 24 enrolled participants were included in the RCT analysis. The LFU analysis comprised 362 participants from non-randomized studies (open-label extension phases, cohort studies, observational studies, and case series), of whom 81 had also contributed to the RCT analyses. As RCTs and non-randomized studies constitute distinct analytical strata, this overlap did not represent double-counting. Where participants received sequential stimulation at more than one anatomical target, each target phase was entered as an independent trial arm without duplicating participant counts. In Tyagi et al. (2019) [50], 6 participants received sequential stimulation at VC/VS and amSTN, each entered as an independent arm in target-specific subgroup analyses (see Section 2.8).

### 2.3. Data Items

The following data items were collected when available:

- General study information: First author, year of publication, study design (RCT vs. non-randomized), intervention duration, length of follow-up (months), and sample size. For non-randomized studies, follow-up duration was categorized as short-term (≤12 months) or long-term (>12 months); response and remission rates were also recorded.
- Patient characteristics: Sex, age at DBS surgery, illness duration, severity of OCD at baseline, and comorbid MDD diagnosis. Patient-level data were collected when available. When not reported, group-level characteristics were extracted.

### 2.4. Endpoints and Sub-Analysis

The primary outcome was OCD symptom severity, measured as mean Y-BOCS score change (points) and percent reduction from baseline. Sub-analyses examined response (≥35% reduction) [51] and remission rates (≤14 Y-BOCS score) [52,53,54]. Patient-level data were prioritized. When unavailable, aggregated data were extracted. All data were manually compiled in a spreadsheet and independently verified by a second reviewer.

### 2.5. Identification of Active Stimulation Sites

Subgroup analyses were conducted using both author-reported target classifications and our team’s anatomical reclassification. Reclassification was performed independently by two evaluators (S. Dufault and S. Patry), with discrepancies resolved by structured consensus discussion. Active site determination was based on electrode trajectory, stereotactic coordinates, active contact localization, electrode geometry, contact configuration, and stimulation parameters, in accordance with established neuroanatomical frameworks and stereotactic atlases [55,56,57,58,59]. Full classification criteria are documented in Table S8. In cases of ambiguity, the volume of tissue activated was estimated using Guide Element^®^ software (version 3.3.0; Boston Scientific, Valencia, CA, USA), and electrode placement was visualized in Brainlab Stimview XT^®^ (version 1.0.0; Brainlab, Munich, Germany) from reported MNI coordinates. When confirmation remained impossible, the original author classification was retained.

### 2.6. Secondary Outcomes

Anxiety, depression, and global functioning were assessed as secondary outcomes. Anxiety was measured using the Hamilton Anxiety Rating Scale (HAM-A) or the State-Trait Anxiety Inventory (STAI). Depression was assessed using the Hamilton Depression Rating Scale (HAM-D), the Montgomery–Åsberg Depression Rating Scale (MADRS), or the Beck Depression Inventory (BDI). Global functioning was assessed using the Global Assessment of Functioning (GAF).

### 2.7. Assessment of Quality Study

Risk of bias (RoB) in RCTs was assessed using the revised Cochrane Risk-of-Bias tool (RoB 2) [60], in accordance with the Cochrane Handbook for Systematic Reviews of Interventions [61], across the following domains: randomization, carryover effects in crossover trials, deviations from the intended intervention, missing data, outcome measurement, and selection of reported results. Non-randomized studies were assessed using the Cochrane ROBINS-I tool [62] across seven domains: pre-intervention (confounding, participant selection), during intervention (misclassification, mismeasurement), and post-intervention (deviations, missing data, outcome measurement, reported results). Each domain was rated as high risk/serious risk, moderate risk/some concerns, low risk, or no information. Two reviewers independently performed all assessments, resolving disagreements by consensus. Certainty of evidence for the primary outcome was evaluated using the Grading of Recommendations, Assessment, Development and Evaluation (GRADE) approach, rating it as high, moderate, low, or very low based on risk of bias, inconsistency, indirectness, imprecision, and publication bias [47,63].

### 2.8. Statistical Analysis

Comprehensive Meta-Analysis (version 2) was used to calculate effect size estimates for the impact of DBS on the Y-BOCS [64]. Effect sizes for the Y-BOCS were expressed as mean differences in absolute score (points) and as mean percentage improvements from baseline. or the primary outcome, mean difference was preferred over Cohen’s d given that all trials used the same outcome measure (Y-BOCS), facilitating clinical interpretability. Separate analyses were performed for RCTs (DBS versus sham stimulation) and non-randomized studies. For non-randomized studies, analyses were conducted at three time points: short-term follow-up (≤12 months), long-term follow-up (>12 months), and last available follow-up.

In addition to the Y-BOCS, analyses evaluated DBS effectiveness on secondary outcomes, namely anxiety, depression, and global functioning (measured using the GAF). For anxiety and depression, effect sizes were calculated using Cohen’s *d*, defined as a standardized mean difference, with 0.2, 0.5, and 0.8 considered as small, medium, and large effects, respectively [65]. Cohen’s *d* was chosen over mean difference because these outcomes were assessed using different scales (e.g., BDI, HAM-A, HAM-D, MADRS, STAI). Effect sizes were considered positive when primary or secondary outcomes improved following DBS.

Effect sizes were estimated as mean differences (primary outcome, Y-BOCS) or Cohen’s *d* (secondary outcomes). When the pre–post correlation was not reported, its value was imputed in accordance with the recommendations of Rosenthal (1991) [66]. Heterogeneity was assessed using Cochran’s Q statistic [67] and its magnitude evaluated with the I^2^ index [68]. Considering that results were mostly heterogeneous, estimates were pooled using a random-effects model, which accounts for between-study variance and permits population-level inference. Between-study variance (τ^2^) was estimated using the DerSimonian–Laird method [69], a well-established and widely used estimator implemented in Comprehensive Meta-Analysis (version 2). Analyses were conducted at the trial-arm level. For studies reporting distinct patient subgroups stimulated at different anatomical targets, each subgroup was entered as an independent arm. For crossover studies in which the same participants received stimulation at multiple targets sequentially, each target phase was entered as an independent arm exclusively into its corresponding target-specific forest plot, consistent with Cochrane guidance on separating multi-arm comparisons into distinct forest plots.

Publication bias was assessed using Egger’s regression test and visual inspection of the funnel plot [70].

To estimate responder and remission rates, additional analyses were conducted. A reduction of ≥35% in Y-BOCS score was used as the criterion for response at last follow-up [51], while a Y-BOCS score of ≤14 was used as the criterion for remission, given that no universally accepted remission threshold exists and published definitions typically range from ≤12 to ≤14 [52,53,54].

To assess the robustness of pooled estimates, two sensitivity analyses were conducted on the non-randomized studies at last follow-up. First, each study was sequentially removed and the analysis was rerun to evaluate the influence of individual studies on the overall effect. Second, trial arms with *n* < 5 were excluded and the analysis was repeated.

To estimate the effect of stimulation site on DBS effectiveness, separate subgroup analyses were performed for each stimulation site, restricted to non-randomized studies at last follow-up, using the mean difference (MD) in Y-BOCS scores as the outcome of interest. Stimulation sites were initially grouped according to the anatomical labels reported by the investigators (e.g., caudate, NAc, vALIC). Because historical target nomenclature was often inconsistent and imprecise, stimulation sites were subsequently reclassified according to the criteria described above. Detailed classification procedures are presented in Supplementary Table S8, and stereotactic coordinates, anatomical stimulation sites, stimulation parameters, and DBS lead models are reported in Supplementary Table S10.

To investigate potential predictors of DBS effectiveness, meta-regression analyses were performed using continuous study-level variables, including age (years), sex (% male), illness duration (years), proportion of patients with comorbid MDD (%), baseline Y-BOCS score, study duration (months), and year of publication. Analyses were restricted to non-randomized studies at last follow-up, with the mean difference in Y-BOCS scores serving as the dependent variable. Additional subgroup analyses were conducted according to study risk of bias. Detailed results are presented in Supplementary Table S7.

## 3. Results

### 3.1. Study Selection

**Figure 1:**
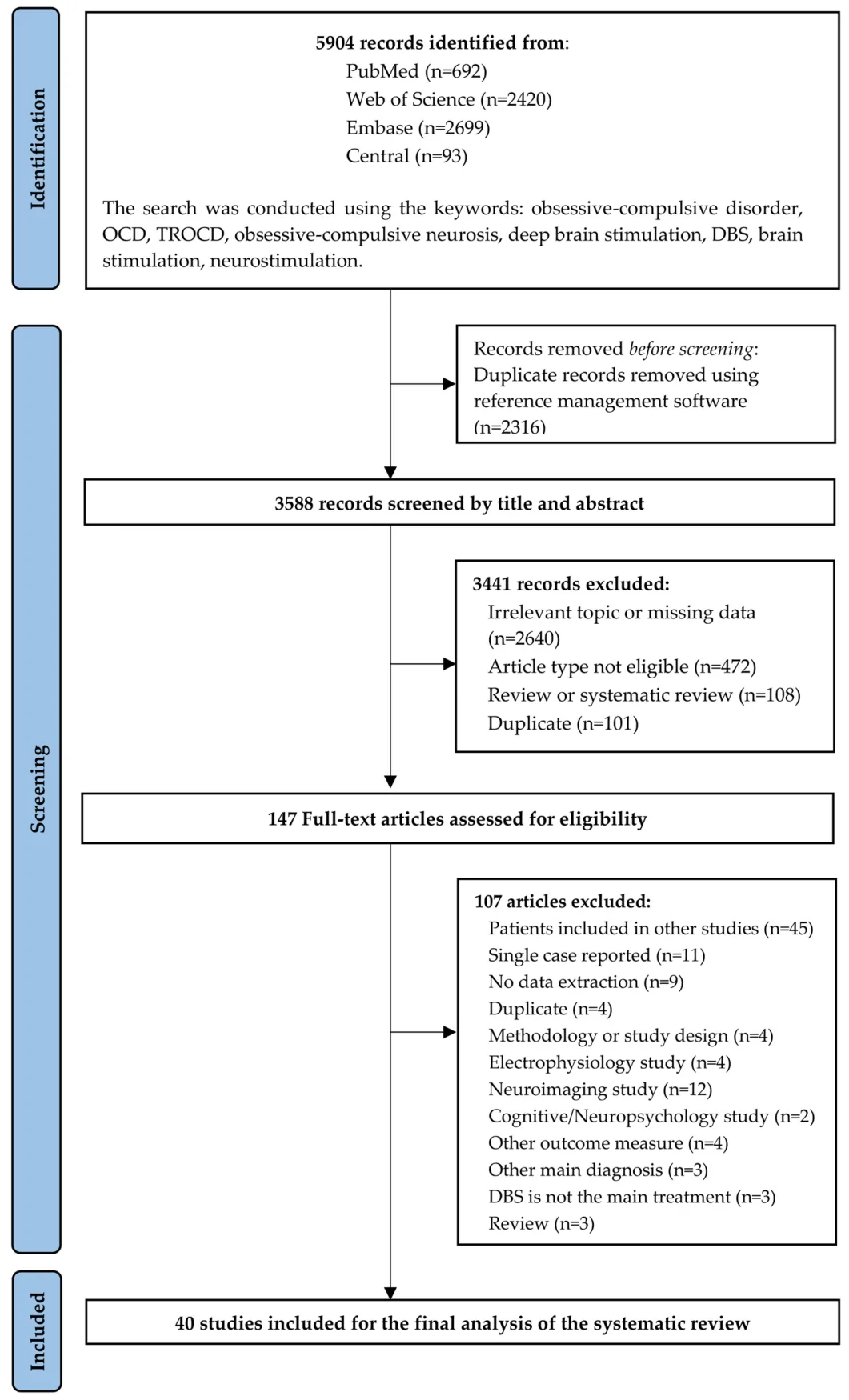
2020 PRISMA Flow chart diagram of studies selection process for systematic reviews and meta-analyses.

Our search strategy yielded 5904 records through July 2025. A total of 2316 duplicate records were removed using EndNote 21 and Zotero. Following title and abstract screening, 3441 records were excluded. Subsequently, 147 full-text articles were assessed for eligibility, of which 107 were excluded for not meeting the predefined inclusion criteria. In total, 40 studies were included in the systematic review and meta-analysis: 8 randomized controlled trials (7 crossover and 1 parallel-group design) enrolling 93 participants, and 32 non-randomized studies enrolling 321 participants. Participant-level exclusions from RCT analyses are detailed in Table 1. The last follow-up analysis comprised 362 participants from non-randomized studies, including open-label extension phases, cohort studies, observational studies, and case series. Figure 1 presents the PRISMA flow diagram summarizing the literature search and study selection process.

### 3.2. Study Characteristics: Demographics and Clinical Profiles

Patient characteristics are presented in Table 1. The analytic sample comprised 83 participants from eight RCTs and 294 participants from 32 non-randomized studies, for a total of 377 adult participants. The number of participants contributing to each pooled analysis varied by analytical stratum and follow-up interval and is reported in the corresponding results sections. Participants had severe OCD at baseline, with a mean age at DBS surgery of 39.61 ± 5.69 years. The mean baseline Y-BOCS score was 33.50 ± 2.14 points, and the mean duration of OCD was 23.12 ± 6.35 years. Mean follow-up duration in non-randomized studies ranged from 6 to 78 months. Psychiatric comorbidities were reported in 25 studies (*n* = 268), with major depressive disorder as the most frequently reported comorbidity, affecting 51% of those with available comorbidity data.

### 3.3. Study Characteristics: Anatomical Stimulation Sites

The most frequently reported DBS targets were located in the limbic territory. In some studies, multiple targets were mentioned, or the same participants may have received stimulation at different sites. Regardless of the targets reported by the investigators, an analysis of the anatomical sites of active stimulation performed by our team revealed that the most frequent targets, based on the number of patients per anatomical site, were the vALIC (*n* = 79) [37,44,50,92], followed by the vALIC-NAc (*n* = 70) [12,37,42,45,76,78,85,87,96], BNST-ALIC (*n* = 49) [42,46,81,84,95], VC/VS (*n* = 39) [76,79,82,86,93,94,97], amSTN (*n* = 33) [50,89,91], slMFB (*n* = 11) [32,35], ITP (*n* = 11) [80,88], NAc (*n* = 8) [81,84,90], ALIC (*n* = 8) [98], and the MD/VA thalamic nuclei (*n* = 4) [83], as well as the caudate nucleus (*n* = 2) [77]. DBS leads were implanted bilaterally in all studies, with the exception of Huff et al. [74] in which stimulation was applied unilaterally to the right NAc (*n* = 7) and the right vALIC-NAc (*n* = 3). Additionally, five patients were implanted unilaterally in other studies [72,76,93]. For further details refer to Supplementary Tables S3 and S10.

### 3.4. Study Characteristics: Outcomes Related to Depression, Anxiety, and Global Functioning

Twenty-three studies (24 arms) assessed the effects of DBS at pre- and postoperative time points on depressive symptoms [35,36,38,42,45,46,71,74,76,79,82,83,85,86,87,88,95,99]. Seven studies assessed anxiety; 13 studies (14 arms) assessed global functioning using GAF [35,38,42,46,71,74,78,79,85,91,96,99,100].

### 3.5. Risk of Bias Analysis

In RCTs, the overall risk of bias (RoB) for randomization, missing outcomes, and selection of reported results was generally low. Similarly, RoB for intervention assignment and outcome measurement was low in most trials. Main concerns arose from insufficient washout or possible carryover effects in DBS-ON/DBS-OFF phases, though none reached critical or high RoB. Among crossover trials, four included a washout period, whereas four lacked such a period or did not assess potential carryover effects [49,50,71,73]. One trial used two 3-week ON and two 3-week OFF periods in constrained random order, likely insufficient for washout and too short for optimal stimulation, possibly contributing to nonsignificant results [71]. One trial without washout reported no significant carryover [36]. Stimulation phases ranged from 3 weeks to 3 months; one trial used a parallel-arm design [75]. In one study, the stimulation target (amSTN) could have compromised allocation concealment if motor side effects were observed [72]. Investigator unblinding was reported in one trial [36]. Deviations from the intended intervention were sometimes attributable to patient choice or adverse events [36,49,50]. Detailed RoB assessments are presented in Supplementary Figure S1.

In non-randomized studies, RoB for intervention classification, deviations, outcome measurement, and selective reporting was mostly low to moderate. Confounding was low in 23% but moderate to serious in 77%, likely reflecting variability in targets, anatomical regions, follow-up duration, comorbidities, baseline severity, and symptom dimensions. Serious RoB from missing data occurred in one study with 50% retention at 24–36 months [80]. Another introduced confounding when two patients initially implanted in the NAc were subsequently reimplanted in the thalamus [83]. In one trial, only DBS responders were included [45]. These findings are consistent with prior systematic review [29]. Detailed RoB assessments are presented in Supplementary Figure S2.

### 3.6. Meta-Analysis Results for Primary Outcome

Randomized controlled trials (RCTs)

**Figure 2:**
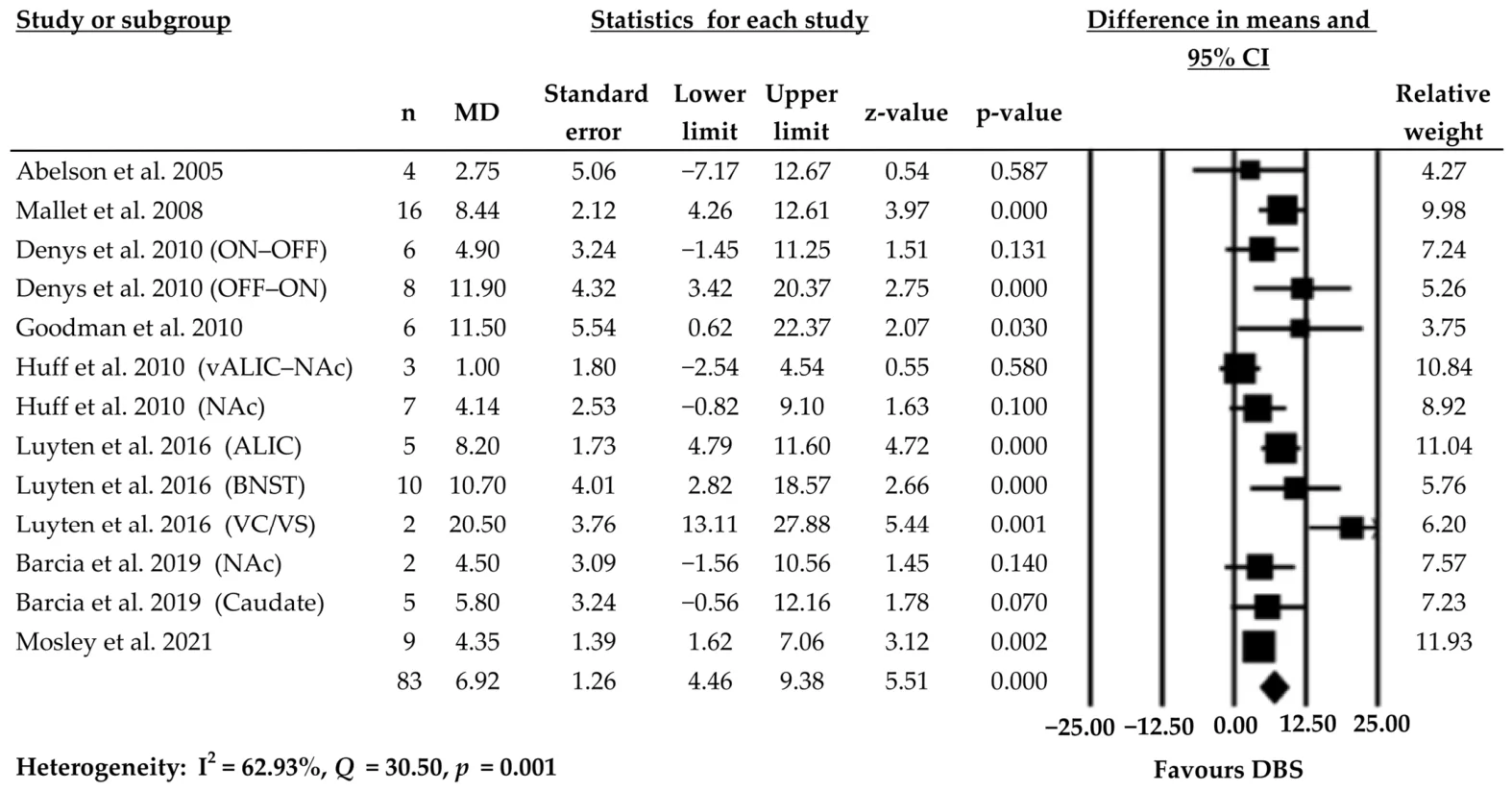
Meta-analysis results for the primary outcome in randomized controlled trials. RCTs comparing active and sham deep brain stimulation, reporting the mean Y-BOCS score change in patients with treatment-resistant obsessive compulsive disorder [36,38,49,71,72,73,74,75]. MD, mean difference.

Across 8 RCTs (*n* = 83) comparing active DBS with sham stimulation (DBS-OFF), pooled analyses demonstrated significant symptom reductions for both primary outcomes. Y-BOCS total score improved by a mean difference of 6.92 points (95% CI: 4.46–9.38; Figure 2; Table 2), with substantial heterogeneity across studies (I^2^ = 62.93%, *p* = 0.001). Percentage improvement, available in 7 of 8 RCTs (*n* = 69; Denys et al. [36] reported absolute score changes only), yielded a pooled reduction of 18.31% (95% CI: 11.42–25.09), with low and non-significant heterogeneity (I^2^ = 28.87%, *p* = 0.170). Funnel plot inspection and Egger’s test indicated no evidence of publication bias for either outcome (Table 2 and Figure S3).

Non-Randomized Studies: Outcomes at Last Follow-Up

**Figure 3:**
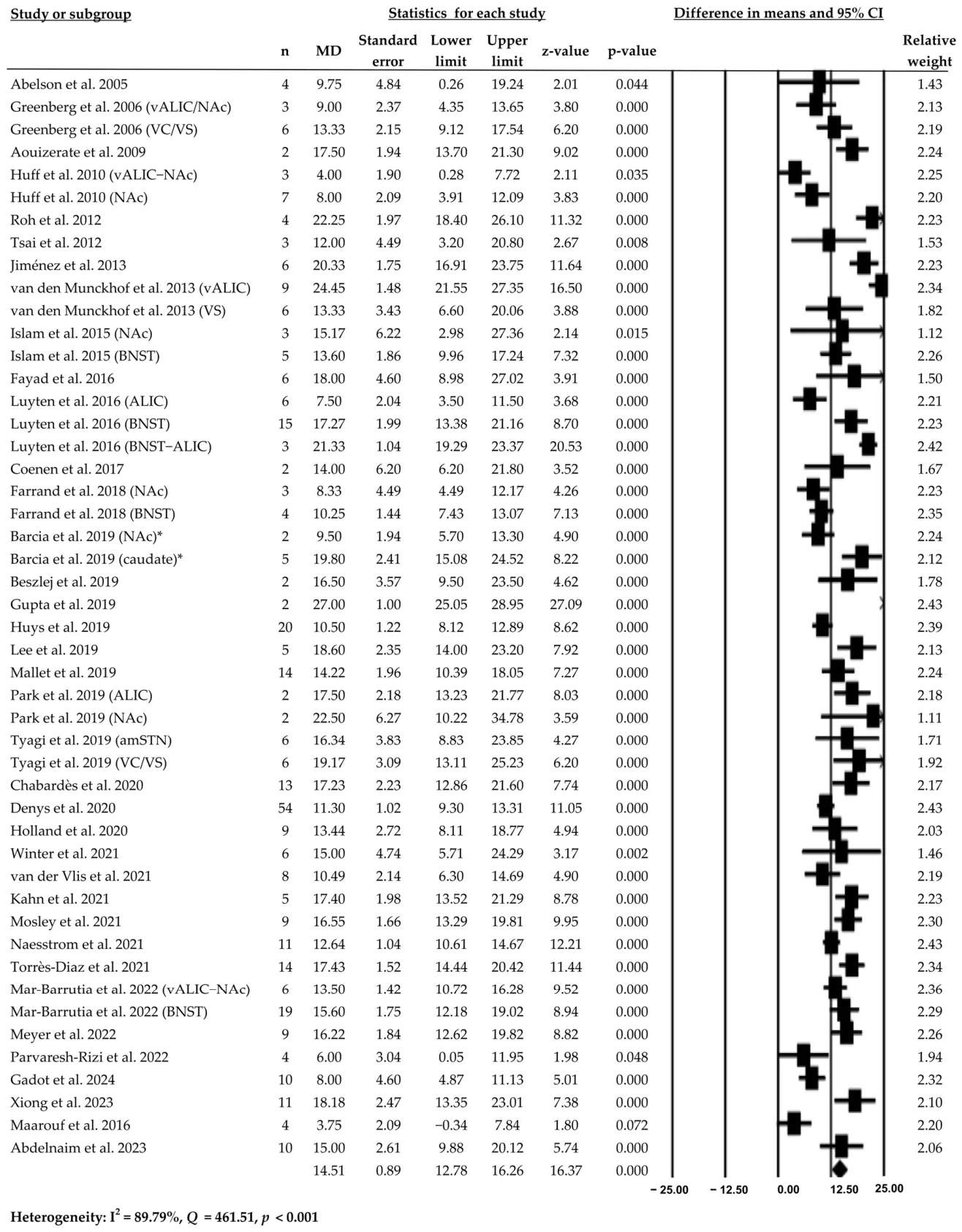
Meta-analysis of Y-BOCS score change at last follow-up in non-randomized studies. Data were pooled from 37 studies (48 trial arms; *n* = 362 participants) [32,35,37,38,42,43,44,45,46,49,50,71,74,75,76,77,78,79,80,81,82,83,86,87,88,89,90,91,92,93,94,95,96,97]. Where participants received stimulation at more than one anatomical target, each arm was analyzed independently [50]. Participant counts reflect unique individuals. MD, mean difference; CI, confidence interval; I^2^, between-study heterogeneity. * Best contacts. The 37 studies include the 32 non-randomized studies (Table 1B) and 5 long-term follow-up publications from RCT cohorts retained as the most complete datasets for their respective cohorts.

In non-randomized studies at last follow-up, significant improvements were observed for both Y-BOCS score and Y-BOCS percent change, with a mean difference of 14.51 points (95% CI: 12.78 to 16.26) and a 43.50% reduction (95% CI: 38.82 to 48.27), respectively (Figure 3; Table 2). Results were heterogeneous for both outcomes. No evidence of publication bias was detected.

Non-Randomized Studies: Outcomes at Short-Term Follow-Up

In non-randomized studies at short-term follow-up, significant improvements were observed for both Y-BOCS score and Y-BOCS percent change, with a mean difference of 13.83 points (95% CI: 11.01 to 16.52) and a 40.22% reduction (95% CI: 32.47 to 47.89), respectively (Table 2). Results were heterogeneous for both outcomes.

Non-Randomized Studies: Outcomes at Long-Term Follow-Up

In non-randomized studies at long-term follow-up, significant reductions in both Y-BOCS score and Y-BOCS percent change were observed, with a mean difference of 17.34 points (95% CI: 14.70 to 20.04) and a 49.81% reduction (95% CI: 42.57 to 57.03), respectively (Table 2). Results were heterogeneous for both outcomes.

### 3.7. Subanalyses

#### 3.7.1. Response and Remission Rates

Among non-randomized studies at last follow-up, 60.21% (95% CI: 52.78–67.17) of participants met responder criteria (Y-BOCS reduction ≥ 35%) [51], with low heterogeneity across studies (I^2^ = 20.74%). Remission (Y-BOCS ≤ 14) was achieved in 36.40% (95% CI: 29.78–43.45) of participants [52,53,54], with similarly low heterogeneity (I^2^ = 12.08%). Detailed response and remission rates are presented in Supplementary Table S4.

#### 3.7.2. DBS Anatomical Stimulation Sites

Subanalyses by stimulation site were conducted using Y-BOCS mean difference data from non-randomized studies at last follow-up, based on both author-reported targets and our team’s anatomical recategorization using active contact location and stereotactic coordinates. Results were heterogeneous across sites under both classification systems (authors: Q = 78.18, *p* < 0.001; recategorization: Q = 59.90, *p* < 0.001).

Using author-reported classifications, the largest improvements were observed for the inferior thalamic peduncle (ITP; 19.70 points), caudate nucleus (18.41 points), caudal VC/VS (17.78 points), and ALIC-NAc (17.54 points), whereas NAc stimulation yielded the smallest improvement (9.7 points). No significant effect was observed for mediodorsal/ventral anterior (MD/VA) thalamic stimulation.

In our anatomical recategorization, the internal capsule and its subdivisions (ALIC, vALIC, and vALIC-NAc), the most frequently reported stimulation sites in this literature, demonstrated comparable effectiveness, with mean differences ranging from 13.1 to 14.7 points (*p* < 0.001). VC/VS and BNST-ALIC sites, located caudally to the VS at the level of the anterior commissure where major limbic and prefrontal projection pathways converge, yielded improvements of 15.3 to 16.1 points. The slMFB, an ascending dopaminergic pathway originating in the ventral tegmentum and projecting to prefrontal regions [101], and the amSTN demonstrated improvements of 15.83 and 15.64 points, respectively. Overall, all stimulation targets except the MD/VA thalamic nuclei were associated with significant therapeutic benefit in TR-OCD, with Y-BOCS improvements ranging from 9.7 to 19.7 points. Detailed results are presented in Table 3.

### 3.8. Sensitivity Analyses

For both the mean difference in Y-BOCS scores and percentage change at last follow-up, leave-one-out sensitivity analysis confirmed the robustness of the findings, as no individual study altered the pooled estimates by more than 2%. Similarly, exclusion of study arms including fewer than five participants yielded results comparable to the primary analysis, with a pooled mean difference in Y-BOCS scores of 14.92 points (95% CI: 13.40 to 16.47) and a 43.61% reduction (95% CI: 39.42 to 47.78) at last follow-up. Detailed results are presented in Supplementary Table S6.

### 3.9. Secondary Outcomes

Using data from non-randomized studies at last follow-up, DBS was associated with large, significant improvements in comorbid anxiety (*d* = 1.1) and depression (*d* = 1.1). Significant improvements in global functioning were also observed (MD = 25.62). Results for all secondary outcomes were heterogeneous across trials. Results are presented in Supplementary Table S5.

### 3.10. Meta-Regression Analyses

Using data from non-randomized studies at last follow-up, meta-regression analyses showed that age, sex ratio, illness duration, proportion of patients with comorbid MDD, baseline OCD severity, and year of publication were not significantly associated with the mean change in Y-BOCS scores following DBS (all *p* > 0.05). In contrast, a significant positive association was observed between follow-up duration and the mean change in Y-BOCS scores following DBS (*p* = 0.042). Detailed results are presented in Supplementary Table S7 and Figure S4.

Subanalyses found no significant differences in treatment effect according to risk of bias: serious risk (3 trial arms; 14.62 points; 95% CI: 12.33 to 16.87; *p* < 0.001), moderate risk (37 trial arms; 15.30 points; 95% CI: 14.71 to 15.85; *p* < 0.001), and low risk (8 trial arms; 14.82 points; 95% CI: 13.43 to 16.19; *p* < 0.001); between-group heterogeneity: Q = 0.60; *p* = 0.736.

Based on the GRADE approach, the overall quality of evidence from RCTs for the primary outcome showed low risk of bias across most domains. Some concerns were related to unblinded raters or unclear blinding procedures, absence or inadequacy of washout periods in crossover designs, and variability in the duration of the randomized phase. No trial was rated at high risk of bias. Certainty was downgraded by one level to moderate. Despite substantial heterogeneity for mean difference in Y-BOCS score and moderate heterogeneity for percentage change, effects consistently favored DBS, with no evidence of publication bias (Egger *p* = 0.133). Inconsistency was not considered grounds for downgrading, as all included RCTs demonstrated greater symptom reduction under active DBS versus sham, with no study reporting an effect in the opposite direction. Per GRADE recommendations, inconsistency should be evaluated on the basis of the direction and magnitude of effects rather than solely on the I^2^ statistic [63]. Although the lower bound of the confidence interval (4.5 points) fell within the minimal clinically important difference range for the Y-BOCS (4–6 points), the effect size remained clinically meaningful.

For non-randomized studies, most trials had low to moderate risk of bias, with three rated as serious. Certainty was rated as low at baseline, downgraded for risk of bias, and upgraded for the very large effect size (MD = 14.5 points; SMD = 3.16), yielding a final rating of low. Despite substantial heterogeneity, effects were consistent in direction across all included studies, the confidence interval was narrow (95% CI: 12.8–16.3 points), and heterogeneity was substantially explained by trial duration (meta-regression, *p* = 0.042) and stimulation target (Q = 59.9, *p* < 0.001). Per GRADE Guidance 35, statistical heterogeneity alone does not warrant downgrading for inconsistency [102]. No evidence of publication bias was detected (Egger *p* = 0.247). For a full account of our grading of the certainty of evidence, see Supplementary Figures S5 and S6.

## 4. Discussion

### 4.1. Main Findings

DBS produces significant and sustained symptom reduction in TR-OCD, with greater improvements associated with longer follow-up duration. In RCTs, active DBS reduced Y-BOCS scores by 6.9 points (18.3%) versus sham, a finding somewhat attenuated relative to previously reported estimates [27], which pooled overlapping samples without isolating the active versus sham comparison. At last follow-up (mean 30.4 months), non-randomized studies demonstrated a 14.5-point reduction (43.5%), with further improvement beyond 12 months, reaching a mean difference of 17.3 points (49.8%). Responder and remission rates at last follow-up were 60% and 36.4%, respectively. Comorbid anxiety, depression (*d* = 1.1, each), and global functioning (MD = 25.6) improved significantly, indicating co-therapeutic improvements extending beyond OCD symptom reduction. Meta-regression identified follow-up duration as the only significant predictor of response (*p* = 0.042), independent of stimulation target.

As a secondary objective, we systematically reviewed and harmonized anatomical stimulation targets across all included studies, reclassifying reported sites based on active contact localization, electrode geometry, and stimulation parameters, independent of the nomenclature used by the original study authors. Pooled mean Y-BOCS improvements ranged from 9.7 to 19.7 points across target-specific subgroups. These differences are reported descriptively and should be interpreted as hypothesis-generating, given that between-subgroup variation may reflect study-level confounders, including differences in follow-up duration, patient selection, center expertise, OCD symptom dimensions, and stimulation parameters, rather than target-specific effects per se.

NAc stimulation produced statistically significant improvements that did not reach the established clinical response threshold of ≥35% Y-BOCS reduction (mean improvement: 9.7 points). MD/VA thalamic stimulation showed no statistically significant benefit based on a single trial arm (MD = 3.79, *p* = 0.072), and this estimate warrants cautious interpretation given the limited evidence base. Targets at the junction of the anterior limb and genu at the level of the AC, where fibers from limbic, reward, and prefrontal control networks converge, were associated with the most favorable outcomes. The caudal VC/VS and BNST-ALIC sites yielded mean Y-BOCS reductions of 15.3 to 16.1 points. The BNST, situated immediately posterior to the AC and a few millimeters caudal to the NAc [39,49,97], lies within this fiber convergence zone. The ITP, connecting the OFC to the ascending reticular system and located approximately 6 mm lateral and 3–5 mm posterior to the AC at 0.5 mm below the AC–PC line [40,80,88], was associated with the largest observed improvement (19.70 points), followed by the caudate nucleus (18.41 points). Both findings are based on two trial arms only and should be considered exploratory pending replication in larger prospective cohorts.

The slMFB and amSTN yielded comparable improvements in total Y-BOCS score (15.83 and 15.64 points, respectively). These targets recruit anatomically distinct pathways: the slMFB constitutes an ascending dopaminergic projection from the ventral tegmentum to prefrontal regions, while amSTN stimulation engages the cortico-subthalamic hyperdirect pathway [101]. Despite comparable aggregate outcomes, Tyagi et al. [50] demonstrated dissociable clinical effects between these targets, suggesting differential modulation of OCD symptom dimensions. The same study showed that the volume of tissue activated (VTA) during amSTN stimulation is centered on the medial-antero-inferior STN border, spreading into the ventral tegmental area through which slMFB fibers also pass, suggesting that off-target slMFB recruitment may partially account for the similar magnitude of total Y-BOCS improvement observed at these sites.

Although anatomically proximate, these targets maintain distinct structural connectivity profiles that may differentially engage fiber populations within OCD therapeutic circuits [10,50,101]. BNST stimulation may recruit up to 35 times more reward- and control-network fibers than NAc stimulation, and ITP stimulation may engage up to four times more reward-network fibers and five times more control-network fibers than BNST stimulation [10]. Because DBS effects are strongest near the active contact at the VTA center [31], these targets are not interchangeable and may differentially contribute to clinical outcomes across symptom dimensions [10,39]. These findings are consistent with Meyer et al. [103,104], who identified two optimal stimulation regions: the central ALIC extending into the caudate, and a postero-ventral AC–PC region encompassing the ITP and BNST. Although a common “striatal region” classification has been proposed to reduce terminological ambiguity in the reporting of VC/VS, NAc, and vALIC targets [40], fiber projections within this region are topographically organized [10], and the VTA is shaped by stimulation parameters (amplitude, pulse width, contact configuration, electrode geometry, and polarity), such that anatomically adjacent sites may engage distinct fiber populations and yield clinically non-equivalent outcomes. Overall, with the exception of MD/VA thalamic nuclei, all anatomical targets demonstrated statistically significant therapeutic effects, though observed effectiveness varied substantially across sites. Our results support the hypothesis that targeting the convergence zone of limbic, reward, and prefrontal control projections posterior to the AC, near the genu of the internal capsule at the vALIC junction [34,35,40,75], may most effectively modulate the dysregulated cortico-striatal connectivity within the CSTC circuits underlying OCD pathophysiology.

### 4.2. Limitations

The principal limitation of this meta-analysis is the small aggregate sample size, which constrained the statistical power of subgroup analyses by stimulation site. Effect estimates for targets represented by few studies, particularly the ITP and MD/VA thalamic nuclei, should be considered exploratory pending replication in larger prospective cohorts.

Substantial heterogeneity in non-randomized studies (I^2^ = 89.79–93.90%) likely reflects the diversity of included designs (open-label trials, cohort studies, and case series), alongside variability in follow-up duration, stimulation targets, anatomical nomenclature [40], and stimulation parameters. In RCTs, moderate heterogeneity (I^2^ = 62.93%) may additionally reflect differences in sham-stimulation control period duration and optimization phase procedures, including the use of unilateral rather than bilateral DBS in one trial [74]. A substantial proportion of between-study variance remained unexplained by available study-level covariates, consistent with heterogeneity driven by participant- or site-level factors. Although sensitivity analyses confirmed robustness and consistent directionality of primary estimates, residual heterogeneity limits the precision of pooled effects and warrants caution in interpreting subgroup findings. Shared participants across subgroups are limited to one crossover trial (Tyagi et al. 2019, *n* = 6) [50] and do not affect primary analyses.

Inconsistencies in anatomical target nomenclature across the included literature posed a classification challenge: labels such as NAc, VC/VS, and vALIC are variably applied to overlapping regions without established anatomical boundaries [40,57,58,105]. Recategorization based on stereotactic coordinates, active contact localization, electrode geometry, and stimulation parameters to estimate the center of the VTA mitigated this limitation. Some assignments may nonetheless remain uncertain where reporting was incomplete [39].

A formal synthesis of adverse events was not performed, as reporting varied substantially across included studies in definitions, classification, and attributability to DBS therapy. Rates of hardware complications, infection, seizure, and serious adverse events documented in prior systematic reviews suggest an acceptable safety profile, although the present analysis does not permit independent safety conclusions. This review was not registered in PROSPERO, although eligibility criteria, outcomes, and analytical procedures were predefined prior to data extraction, consistent with the methodological requirements of systematic reviews, and we acknowledge the absence of prospective registration as a limitation. The search was restricted to English-language publications, and non-English studies were not retrieved, which may have introduced language bias and potentially underrepresented contributions from non-Anglophone clinical sites.

### 4.3. Implications

Greater improvement with longer follow-up argues against early discontinuation, and patient counseling should reflect realistic trajectories beyond the first year, as outcomes may depend on iterative parameter adjustments and neural adaptation to sustained stimulation. Co-improvement of comorbid anxiety and depression suggests DBS is particularly suited for patients with complex, multi-domain presentations. Given that symptom onset typically occurs in adolescence yet DBS access often comes more than a decade later [5], and that most patients have by then exhausted available pharmacological and behavioral treatment options, these findings support broader access for carefully selected patients.

Although RCTs provide the strongest evidence, their application in TR-OCD faces structural constraints: small eligible populations, few specialized centers, and patient access to treatment outside trials reduce willingness to accept sham randomization. These constraints should temper recommendations that treat randomized designs as straightforwardly achievable. Non-randomized studies therefore have a justified and necessary role in characterizing real-world effectiveness, and low-certainty evidence has informed clinical guidelines in comparably severe neuropsychiatric conditions [106]. An international prospective registry [107] could harmonize methodologies and enable large-scale analyses across centers.

Caudal VC/VS, BNST–ALIC, and ITP demonstrated the most favorable outcomes and represent priority targets for future investigation. Evidence from a single crossover trial (*n* = 6) suggests that different targets engage distinct fiber pathways with potentially dissociable effects on OCD symptom dimensions [50]. Whether pre-implantation symptom profiling should guide target selection remains an open question requiring prospective investigation. Collaborative research integrating tractography, connectomics, and patient-specific neuroimaging is needed to refine targeting precision and standardize anatomical nomenclature.

## 5. Conclusions

This meta-analysis supports the use of DBS for TR-OCD, with therapeutic effectiveness exceeding a 17-point (~50%) Y-BOCS reduction beyond 12 months and over one-third of patients achieving remission at last follow-up. Targets associated with the most favorable outcomes converge at the anterior limb–genu junction at the level of the AC, where limbic, reward, and prefrontal fiber pathways intersect, though stimulation targets are not therapeutically equivalent. Collaborative efforts integrating standardized anatomical reporting and patient-specific neuroimaging are needed to consolidate the evidence base and optimize clinical outcomes.

## Figures and Tables

**Table 1:** Characteristics of included studies. (A) Randomized controlled trials (RCTs). (B) Non-randomized studies.

**(A) Study RCT**	**Year**	***n***	**Male (%)**	**Age at Surgery (Years)**	**OCD Duration (Years)**	**Follow-Up (Months)**	**Comorbidity MDD (%)**	**Stimulation Target Site (by Authors)**	**Anatomical Structure Stimulated**
Abelson et al. [71]	2005	4	50	40.3	22.5	12.9	75	ALIC	vALIC
Mallet et al. [72]	2008	17	63.5	43.8	30.3	10	50	amSTN	amSTN
Denys et al. [36]	2010	16	56.3	42	29	21	37.5	NAc	vALIC
Goodman et al. [73]	2010	6	33.3	36.2	24	12	100	VC/VS	VC/VS
Huff et al. [74]	2010	10	60	36.3	22.2	12	60	vALIC-NAc	vALIC-NAc
Luyten et al. [49]	2016	24	50	41.4	NR	77.1	NR	ALIC, BNST, VC/VS	ALIC, BNST-ALIC
Barcia et al. [38]	2019	7	43	36.3	25.3	19	43	NAc, Caudate	NAc, Caudate
Mosley et al. [75]	2021	9	56	47.7	38	15	67.7	BNST	BNST-ALIC
Of 93 patients enrolled across 8 RCTs, 83 were included in the meta-analysis. Mallet et al. [72]: 1 of 17 patients did not complete the randomized phase (*n* = 16 analyzed). Denys et al. [36]: 2 of 16 did not complete the crossover DBS-ON/OFF period (*n* = 14 analyzed). Luyten et al. [49]: 17 of 24 enrolled patients participated in the randomized phase (*n* = 17 analyzed). The remaining 7 participated in the open-label phase only and were included in the last follow-up analyses. No participants were excluded from the remaining five RCTs.									
**(B) Study (Non-Randomized Studies)**	**Year**	***n***	**Male (%)**	**Age at Surgery (Years)**	**OCD Duration (Years)**	**Follow-Up (Months)**	**Comorbidity MDD (%)**	**Stimulation Target Site (by Authors)**	**Anatomical Structure Stimulated**
Greenberg et al. [76]	2006	10	60	35.3	22.5	30.7	80	vALIC-NAc, VC/VS	vALIC-NAc, VC/VS
Aouizerate et al. [77]	2009	2	100	51	34	15	100	Caudate	Caudate
Roh et al. [78]	2012	4	75	33.8	16.8	24	75	VC/VS	NAc
Tsai et al. [79]	2012	4	100	25.5	8.3	15	75	VC/VS	VC/VS
Jiménez et al. [80]	2013	6	50	34.7	16.2	24	0	ITP	ITP
van den Munckhof [37]	2013	16	56.3	42	29	50	38	vALIC, vALIC-NAc	vALIC, vALIC-NAc
Islam et al. [81]	2015	8	62.5	43.6	29.3	33.8	63	NAc, BNST	NAc, BNST
Fayad et al. [82]	2016	6	33.3	36.2	24	114	100	VC/VS	VC/VS
Maarouf et al. [83]	2016	4	25	39.3	23.5	11.5	50	MD/VA thalamus	MD/VA thalamus
Coenen et al. [32]	2017	2	100	41	29	12	NR	slMFB	slMFB
Farrand et al. [84]	2018	7	43	46.6	25	30.9	29	NAc, BNST	NAc, BNST
Beszlej et al. [85]	2019	2	50	33	21	6	0	ALIC-NAc	ALIC-NAc
Gupta et al. [86]	2019	2	0	46.5	23	42	NR	VC/VS	vALIC
Huys et al. [87]	2019	20	50	43.2	26.1	12	NR	vALIC-NAc	vALIC-NAc
Lee et al. [88]	2019	5	40	32.4	16.2	49.8	NR	ITP	ITP
Mallet et al. [89]	2019	14	57	43.8	30.3	40.9	50	amSTN	amSTN
Park et al. [90]	2019	4	75	35.8	11.3	52.5	NR	ALIC, NAc	ALIC, NAc
Tyagi et al. [50]	2019	6	83	45.5	24.2	14	NR	amSTN, VC/VS	amSTN, vALIC
Chabardès et al. [91]	2020	19	38.5	40.3	18.5	24	8	amSTN	amSTN
Denys et al. [92]	2020	70	31	41.7	25	12	43	vALIC	vALIC
Holland et al. [93]	2020	9	56	40.2	NR	54.7	78	VC/VS	VC/VS
Kahn et al. [94]	2021	5	80	39.2	23	25.5	100	VC/VS	VC/VS
Naesström et al. [95]	2021	11	57.1	38	19.8	12	0	BNST	BNST-ALIC
Díaz et al. [96]	2021	14	64	42.4	21.1	35.9	NR	ALIC-NAc	ALIC-NAc
van der Vlis et al. [97]	2021	8	25	46	29.5	26.3	50	VC/VS	VC/VS
Winter et al. [98]	2021	6	67	39.7	22.2	78	67	BNST-ALIC	ALIC
Mar-Barrutia et al. [42]	2022	25	33.3	49.8	32.6	76	40	vALIC-NAc, BNST	vALIC-NAc, BNST-ALIC
Meyer et al. [35]	2022	9	55.6	32.2	23.4	24.1	NR	slMFB	slMFB
Parvaresh-Rizi et al. [43]	2022	4	0	32	17	11	0	vALIC-NAc	vALIC-NAc
Abdelnaim et al. [46]	2023	10	50	37.4	19.9	9.6	50	BNST	BNST-ALIC
Xiong et al. [45]	2023	13	38.5	31.9	13.6	NR	NR	ALIC-NAc	vALIC-NAc
Gadot et al. [44]	2024	10	NR	37	18.3	6	80	vALIC	vALIC
NR, not reported. Values are reported as means unless otherwise specified. A total of 16 patients from van den Munckhof et al. [37] were also included in the multicenter study from Denys et al. [92]. For the LFU analysis, these 16 patients were excluded from Denys et al. [92] to avoid duplicate observations. Fayad et al. [82] represents the long-term follow-up of the RCT conducted by Goodman et al. [73], and Mallet et al. [89] represents the long-term follow-up of the RCT by Mallet et al. [72]. Participants from Chabardès et al. [91] and Mallet et al. [72,89] were enrolled in STOC studies. Because 6 participants from Chabardès et al. [91] were also included in Mallet et al. [89], they were excluded from the LFU analyses to avoid duplication.									

**Table 2:** Pooled DBS effects on OCD symptom severity across RCTs and non-randomized studies.

Outcomes	*n* Pre-Op	*n* Analyzed	Trial Arms	Mean Difference	95% CI	*p*-Value	Heterogeneity	Publication Bias
Randomized Controlled Trials (DBS vs. sham-stimulation/DBS-OFF)								
Y-BOCS score	93	83	13	6.92	4.46 to 9.38	<0.001	*Q* = 30.50; *p* = 0.001; I^2^ = 62.93%	t = 1.68; *p* = 0.133
Y-BOCS %	77	69	11	18.31	11.42 to 25.09	<0.001	*Q* = 14.09; *p* = 0.170; I^2^ = 28.87%	t = 0.80; *p* = 0.427
Non-Randomized Studies: Outcomes at Last Follow-Up								
Y-BOCS score	384	362	48	14.51	12.78 to 16.26	<0.001	*Q* = 461.51; *p* < 0.001; I^2^ = 89.79%	t = 1.22; *p* = 0.247
Y-BOCS %	388	359	47	43.50	38.82 to 48.27	<0.001	*Q* = 354.01; *p* < 0.001; I^2^ = 87.30%	t = 1.41; *p* = 0.178
Non-Randomized Studies: Outcomes at Short-Term Follow-Up								
Y-BOCS score	270	258	28	13.83	11.01 to 16.52	<0.001	*Q* = 441.47; *p* < 0.001; I^2^ = 93.90%	—
Y-BOCS %	290	278	29	40.22	32.47 to 47.89	<0.001	*Q* = 377.76; *p* < 0.001; I^2^ = 92.62%	—
Non-Randomized Studies: Outcomes at Long-Term Follow-Up								
Y-BOCS score	203	174	25	17.34	14.70 to 20.04	<0.001	*Q* = 262.89; *p* < 0.001; I^2^ = 90.92%	—
Y-BOCS %	178	149	26	49.81	42.57 to 57.03	<0.001	*Q* = 262.89; *p* < 0.001; I^2^ = 90.90%	—

Note: Analyses were conducted at the trial-arm level. For studies in which participants received stimulation at more than one anatomical target, each arm was analyzed independently and participant counts reflect unique individuals. The number of participants varied across analyses, reflecting differences in outcome reporting across included studies.

**Table 3:** DBS therapeutic effects on OCD symptoms improvement measured by the Y-BOCS. (A) DBS effects by anatomical target as reported by study authors. (B) DBS effects by anatomical target recategorized by our research team based on anatomical stimulation sites as reported in the included studies.

DBS-Target	Trial Arms	Mean Difference	95% CI	*p*-Value
**(A)**				
NAc	5	9.70	6.81 to 12.58	<0.001
vALIC-NAc	6	9.41	6.29 to 12.55	<0.001
vALIC	3	14.63	5.53 to 23.72	0.002
ALIC	3	11.80	4.36 to 19.17	0.002
ALIC-NAc	3	17.54	15.08 to 19.90	<0.001
VC/VS	8	17.78	13.90 to 21.58	<0.001
Caudate nucleus	2	18.41	15.42 to 21.39	<0.001
BNST	7	14.20	12.24 to 16.11	<0.001
BNST-ALIC	1	15.04	5.71 to 24.26	0.002
MD/VA Thalamus	1	3.79	−0.30 to 7.76	0.072
ITP	2	19.70	17.01 to 22.49	<0.001
slMFB	2	15.83	12.60 to 19.09	<0.001
amSTN	3	15.64	12.89 to 18.30	<0.001
**(B)**				
NAc	5	9.70	6.81 to 12.58	<0.001
vALIC-NAc	10	13.11	9.56 to 16.60	<0.001
vALIC	5	14.70	7.82 to 21.58	<0.001
ALIC	3	13.10	5.69 to 20.51	0.003
VC/VS	7	16.12	9.90 to 22.42	<0.001
Caudate nucleus	2	18.37	15.40 to 21.39	<0.001
BNST-ALIC	8	15.31	12.13 to 18.61	<0.001
MD/VA Thalamus	1	3.79	−0.30 to 7.76	0.072
ITP	2	19.70	17.01 to 22.49	<0.001
slMFB	2	15.83	12.60 to 19.09	<0.001
amSTN	3	15.64	12.89 to 18.30	<0.001

## Data Availability

All data analyzed in this study were extracted from previously published studies and are included in the article and its Supplementary Materials. Additional information is available from the corresponding authors upon reasonable request.

## Supplementary Materials

The following supporting information can be downloaded at: https://www.mdpi.com/article/10.3390/brainsci16080789/s1, Table S1: Literature search strategy; Table S2: Number of records retrieved by database; Table S3: Number of patients per stimulation target; Table S4: Response and remission rates to DBS therapy measured by the Y-BOCS; Table S5: Effects of DBS on Y-BOCS scores in non-randomized studies with ≥5 participants at last follow-up; Table S6: Effects of DBS on comorbid affective symptoms and global functioning at last follow-up; Table S7: Meta-regression of study-level predictors of DBS effectiveness; Table S8: Methodology for target definition and classification criteria; Table S9: Detailed characteristics of included studies; Table S10: Summary of studies reporting stereotactic coordinates, anatomical sites of stimulation, stimulation parameters and DBS-lead models; Table S11: PRISMA 2020 Main Checklist; Figure S1: Risk of bias assessment for randomized controlled trials using the Cochrane RoB 2 tool; Figure S2: Risk of bias assessment for non-randomized studies using the Cochrane ROBINS-I tool; Figure S3: Publication bias assessment for the mean difference in Y-BOCS scores in non-randomized studies at last follow-up; Figure S4: Meta-regression of follow-up duration and mean difference in Y-BOCS Scores in non-randomized studies at last follow-up; Figure S5: GRADE assessment of the certainty of evidence from randomized controlled trials evaluating the efficacy of DBS based on the mean difference in Y-BOCS scores in TR-OCD; Figure S6: GRADE assessment of the certainty of evidence from non-randomized studies evaluating the effectiveness of DBS based on the mean difference in Y-BOCS scores in TR-OCD; Figure S7: DBS targets and stereotactic coordinates of stimulation sites based on anatomical reconstruction.

## Abbreviations

The following abbreviations are used in this manuscript:

ALIC	Anterior limb of internal capsule
amSTN	Antero-medial subthalamic nucleus
ATR	Anterior thalamic radiation
BNST	Bed nucleus of the stria terminalis
CSTC	Cortico-striato-thalamo-cortical
ITP	Inferior thalamic peduncle
LFU	Last follow-up
MFB	Medial forebrain bundle
MD/VA	Thalamic nuclei, medial-dorsal/ventral-anterior
NAc	Nucleus accumbens
RoB	Risk of bias
slMFB	Superolateral medial forebrain bundle
TR-OCD	Treatment-resistant obsessive compulsive disorder
vALIC	Ventral anterior limb of the internal capsule
VC/VS	Ventral capsule/ventral striatum
VTA	Volume of tissue activated
Y-BOCS	Yale–Brown Obsessive Compulsive Scale
