# Supplementary material for: Effectiveness of Deep Brain Stimulation for Treatment-Resistant OCD: Systematic Review of Anatomical Targets and Meta-Analysis of Randomized and Non-Randomized Studies
Source: Brain Sci. 2026 Jul 27;16(8):789. doi: 10.3390/brainsci16080789 (PMC13511004; doi:10.3390/brainsci16080789)
Supplement: Supplementary file 1 [file brainsci-16-00789-s001.zip › Supplementary Materials (version for publication)_Brain Sciences_July 25 2026.pdf]

Effectiveness of deep brain stimulation for treatment-resistant OCD: Systematic review of anatomical targets and meta-analysis of randomized controlled trials and non-randomized studies

Supplementary materials

Supplementary Tables

Table S1. Literature search strategy

Table S2. Number of records retrieved by database

Table S3. Number of patients per stimulation target

Table S4. Response and remission rates to DBS therapy measured by the Y-BOCS

Table S5. Effects of DBS on Y-BOCS scores in non-randomized studies with ≥ 5 participants at last follow-up

Table S6. Effects of DBS on comorbid affective symptoms and global functioning at last follow-up

Table S7. Meta-regression of study-level predictors of DBS effectiveness

Table S8. Methodology for target definition and classification criteria

Table S9. Detailed characteristics of included studies

Table S10. Summary of studies reporting stereotactic coordinates, anatomical sites of stimulation, stimulation parameters and DBS-lead models

Table S11. PRISMA 2020 Main Checklist

Supplementary Figures

Figure S1. Risk of bias assessment for randomized controlled trials using the Cochrane RoB 2 tool

Figure S2. Risk of bias assessment for non-randomized studies using the Cochrane ROBINS-I tool

Figure S3. Publication bias assessment for the mean difference in Y-BOCS scores in non-randomized studies at last follow-up

Figure S4. Meta-regression of follow-up duration and mean difference in Y-BOCS Scores in non-randomized studies at last follow-up

Figure S5. GRADE assessment of the certainty of evidence from randomized controlled trials evaluating the efficacy of DBS based on the mean difference in Y-BOCS scores in TR-OCD

Figure S6. GRADE assessment of the certainty of evidence from non-randomized studies evaluating the effectiveness of DBS based on the mean difference in Y-BOCS scores in TR-OCD

Figure S7. DBS targets and stereotactic coordinates of stimulation sites based on anatomical reconstruction

Search strategy and results

PubMed, Web of Science, EMBASE and CENTRAL were systematically searched from inception (no lower date limit) through July 20th, 2025, in accordance with PRISMA guidelines.

Table S1. Literature search strategy

Database	Coverage Period	Search Date	Search Strategy	Applied Limits
**PubMed/MEDLINE**	From inception to July 2025	July 20th, 2024	("Obsessive-Compulsive Disorder"[Mesh] OR "OCD"[All Fields] OR "Obsessive-Compulsive Neurosis") AND ("Deep Brain Stimulation"[Mesh] OR "DBS" OR "Brain Stimulation" OR "Neurostimulation")	Humans, English
**Web of Science (Clarivate)**	From inception to July 2025	July 20th, 2024	("OCD" OR "Obsessive-Compulsive Disorder") AND ("Deep Brain Stimulation" OR "DBS")	Humans, English
**EMBASE (Ovid)**	From inception to July 2025	July 20, 2025	("Obsessive Compulsive Disorder" OR "OCD").ti,ab,kw. AND ("Deep Brain Stimulation" OR "DBS" OR "Neurostimulation").ti,ab,kw.	Humans, English
**CENTRAL -** Cochrane Central Register of Controlled Trials	From inception to July 2025	July 3, 2026	Title: "obsessive-compulsive disorder" OR "OCD" OR "obsessive compulsive neurosis"; Abstract: "deep brain stimulation" OR "DBS" OR "neurostimulation".	

Search terms were adapted to the syntax and indexing vocabulary of each database (e.g., MeSH for PubMed, Entree for Embase, Title, Abstract and Keywords for CENTRAL).

The following explicit search term were used in PubMed (Medline), Web of Science : ("obsessive-compulsive disorder"[Mesh] OR "OCD" OR "TR-OCD" OR "TR-OCD" OR "obsessive compulsive neurosis" OR "obsessive-compulsive neurosis”) AND ("Deep Brain Stimulation"[Mesh] OR "DBS" OR "brain stimulation" OR "neurostimulation").

<u>**EMBASE:**</u>

Period: 1960 to July 20th, 2025.

1: exp obsessive compulsive disorder/ 55558 text results

2: (obsessive compulsive disorder or OCD).ti,ab. 29144 text results

3: 1 or 2 60987 text results

4: exp deep brain stimulation/62593 text results

5: (deep brain stimulation or DBS).ti,ab. 37474 text results

6: neurostimulation*.ti,ab. 7086 text results

7: 4 or 5 or 6 79493 text results

8: 3 and 7 3186 text results

9: limit 8 to (human and English language) 2820 text results

Table S2. Number of records retrieved by database

Databases	Citations
PubMed (including Medline)	692
Web of Science	2420
Embase (via Ovid)	2699
CENTRAL	93
**Total of records identified**	**5904**

Table S3. Number of patients per DBS target: (A) RCTs; (B) Non-randomized studies (Y-BOCS %/score).

A

RCT	Total participants	Participants analyzed	Target described by authors	Anatomical site of active stimulation
Denys et al. (2010)	16	14	NAc	vALIC
Huff et al. (2010)	7	7	Right NAc	Right NAc
Barcia et al. (2019)	2	2	NAc	NAc
Goodman et al. (2010)	6	6	VC/VS	VC/VS
Luyten et al. (2016)	3	2	VC/VS	BNST-ALIC
Mallet et al. (2008)	17	16	amSTN	amSTN
Luyten et al. (2016)	15	10	BNST	BNST-ALIC
Mosley et al. (2021)	9	9	BNST	BNST-ALIC
Abelson et al. (2005)	4	4	ALIC	vALIC
Luyten et al. (2016)	6	5	ALIC	ALIC
Barcia et al. (2019)	5	5	Caudate	Caudate
Huff et al. (2010)	3	3	Right vALIC-NAc	Right vALIC-NAc

B

Non-Randomized Studies	Total participants	Participants analyzed	Target according to authors	Anatomical site of active stimulation
Greenberg et al. (2006)	6	6	VC/VS	VC/VS
Roh et al. (2012)	4	4	VC/VS	vALIC-NAC
Fayad et al. (2016)	6	6	VC/VS	VC/VS
Gupta et al. (2019)	2	2	VC/VS	VC/VS
Tyagi et al (2019)	6	6	VC/VS	vALIC
Holland et al. (2020)	9	9	VC/VS	VC/VS
Kahn et al. (2020)	5	5	VC/VS	VC/VS
van der Vlis et al. (2021)	8	8	VC/VS	VC/VS
Tsaï et al. (2012)	4	3	VC/VS	VC/VS
Greenberg et al. (2006)	3	3	vALIC-NAc	vALIC-NAc
van den Munckhof et al. (2013)	6	6	vALIC-NAc	vALIC-NAc
Huys et al. (2019)	20	20	vALIC-NAc	vALIC-NAc
Mar-Barrutia et al. (2022)	6	6	vALIC-NAc	vALIC-NAc
Parvaresh-Rizi et al. (2022)	4	4	vALIC-NAc	vALIC-NAc
Islam et al. (2015)	5	5	BNST	BNST-ALIC
Farrand et al. (2018)	4	4	BNST	BNST-ALIC
Naesström et al. (2021)	11	11	BNST	BNST-ALIC
Mar-Barrutia et al. (2022)	19	19	BNST	BNST-ALIC
Abdelnaim et al. (2023)	11	10	BNST	BNST-ALIC
Islam et al. (2015)	3	3	NAc	NAc
Farrand et al. (2018)	3	3	NAc	NAc
Park et al. (2019)	2	2	NAc	NAc
van den Munckhof et al. (2013)	9	9	vALIC	vALIC
Denys et al. (2020)	70	54	vALIC	vALIC
Gadot et al. (2024)	10	10	vALIC	vALIC
Mallet et al. (2019)	14	14	amSTN	amSTN
Tyagi et al. (2019)	6	6	amSTN	amSTN
Chabardès et al. (2020)	19	13	amSTN	amSTN
Coenen et al. (2017)	2	2	slMFB	slMFB
Meyer et al. (2022)	9	9	slMFB	slMFB
Beszlej et al. (2019)	2	2	ALIC-NAc	vALIC-NAc
Xiong et al. (2023)	13	11	ALIC-NAc	vALIC-NAc
Jiménez et al. (2013)	6	6	ITP	ITP
Lee et al. (2019)	5	5	ITP	ITP
Park et al. (2019)	2	2	ALIC	ALIC
Aouizerate et al. (2009)	2	2	Caudate	Caudate
Winter et al. (2021)	6	6	BNST-ALIC	ALIC
Torres Diaz et al. (2021)	14	14	NAc, VS, I.C.	vALIC-NAc
Maarouf et al. (2016)	4	4	MD/VA thalamus	MD/VA thalamus

Table S4. Response and remission rates to DBS therapy measured by the Y-BOCS.

Outcomes	*n* analyzed	Trial arms	Mean difference	95% CI	*p*-value	Heterogeneity
Responder rate	303	45	60.21	52.78 to 67.17	0.007	$Q = 55.48$; $p = 0.115$; $I^2 = 20.74$
Remission rate	278	43	36.40	29.78 to 43.45	< 0.001	$Q = 47.76$; $p = 0.249$; $I^2 = 12.08$

Table S5. Effects of DBS on the Y-BOCS in non-randomized studies at last follow-up with ≥ 5 patients at last follow-up

Outcome	Trial arms	Outcome	95% CI	*p*-value	Heterogeneity
Non-Randomized Studies: Outcomes at the Last Follow-Up					
Mean difference Y-BOCS score change	30	14.92	13.40 to 16.47	<0.001	$Q = 148.21$; $p < 0.001$; $I^2 = 80.39\%$
Y-BOCS % of change	30	43.61	39.42 to 47.78	<0.001	$Q = 117.63$; $p < 0.001$; $I^2 = 75.28\%$

Table S6. Effects of DBS on comorbid affective symptoms and global functioning at last follow-up

Outcomes	Trial arms	Cohen's *d*	95 % CI	*p*-value	Heterogeneity
Anxiety†	7	1.1	0.72 to 1.56	< 0.001	$Q = 13.21$; $p = 0.04$; $I^2 = 54.69\%$
Depression†	24	1.1	0.80 to 1.26	< 0.001	$Q = 63.81$; $p < 0.001$; $I^2 = 64.02\%$
**Outcome**	**Trial arms**	**Mean difference**	**95 % CI**	***p*-value**	**Heterogeneity**
Functioning† (GAF)	14	25.62	22.18 to 29.03	< 0.001	$Q = 75.01$; $p < 0.001$; $I^2 = 82.69\%$

†Graat et al. (2021) contributed data for secondary outcomes only and was not included in the primary Y-BOCS analyses due to participant overlap with Denys et al. (2010) and Liebrand et al. (2019).

Table S7. Meta-regression of study-level predictors of DBS effectiveness

Variables	Results
Age	Slope = 0.095; 95% CI: −0.202 to 0.393; *p* = 0.530
Sex ratio	Slope = 0.033; 95% CI: −0.042 to 0.108; *p* = 0.387
Duration of illness	Slope = −0.028; 95% CI: -0.306 to 0251; *p* = 0.844
Ratio of patients with MDD	Slope = - 0.0001; 95% CI: −0.059 to 0.058; *p* = 0.999
Trial follow-up duration	Slope = 0.063; 95% CI: 0.002 to 0.124; ***p* = 0.042**
Severity of OCD symptoms at baseline	Slope = 0.427; 95% CI: −0.268 to 1.122; *p* = 0.229
Year of publication	Slope = 0.101; 95% CI: −0.239 to 0.442; *p* = 0.560

MDD, major depressive disorder; OCD, obsessive compulsive disorder.

Supplementary Methods: Target Definition and Classification Methodology

The methodology used for target assignment is described above. Detailed target definitions, study classification, and stereotactic information are presented in Tables S8–S10. Target assignment was based on stereotactic coordinates, active contact localization, DBS lead geometry, stimulation parameters, and anatomical descriptions reported by the original authors. When required, anatomical reconstructions and volume of tissue activated (VTA) simulations were performed using Stimview XT® and Guide XT®. Classification criteria were adapted from established neuroanatomical frameworks and stereotactic atlases.

Table S8. Methodology for target definition and classification criteria

Targets	Neuroanatomical References for Target Classification	Anatomical Description and Targeting Considerations of DBS Targets	Criteria for Classification of Stimulation Site and Methodology	Mean coordinates in millimeters (relative to the AC-PC midline)		
				X	Y	Z
NAc	Mai (2004, 2015); Morel (2007); Mavridis (2014); Lucas-Neto et al. (2013); Neto et al. (2008)	Part of the ventral striatum located at the junction of the caudate head and putamen. The posterior NAc is the principal DBS target in OCD. Located inferior to the vALIC and anterior/inferior to the AC. Compared with the VC/VS, the NAc target is slightly more rostral and ventral in stereotactic space.	Active contacts located below the vALIC, anterior to the AC, inferior to the AC-PC plane, and within MNI-defined NAc boundaries. Distal lead tip located within the NAc core or shell.	6 to 8	3 to 5	−4 to −6
vALIC	Mai (2004, 2015)	Ventral portion of the anterior limb of the internal capsule containing fronto-thalamic and cortico-striato-thalamo-cortical white matter fibers connecting orbitofrontal and prefrontal cortices with subcortical structures. Active contacts were confined to the vALIC without NAc involvement.	Active contacts and VTA localized within the ventral ALIC white matter, without NAc involvement. Classification was based on stereotactic coordinates and active contact localization.	11	4 to 5 ant. border	3
vALIC–NAc		Electrode trajectory traversing both the vALIC and NAc, with distal contacts located in the NAc and superior contacts within the vALIC.	Simultaneous stimulation of the vALIC and NAc through active contacts located in both structures.	9 to 13	4 to 9	−2 to −4
VC/VS	Mai (2004, 2015); Morel (2007)	Lies between NAc, ALIC, and AC. For our reclassification, the VC/VS region was defined as the ventro-posterior portion of the ventral capsule, located below the vALIC at the genu of the internal capsule, at the AC and encompassing the ventral caudal striatum. Rostral VC/VS (MNI: $x = 12 \pm 1$, $y = 8 \pm 1$, $z = -3 \pm 1$) was reclassified in this study as the vALIC-NAc site, given its anatomical position in the rostral ventral striatum, inferior to AC–PC and adjacent to vALIC.	Classification was based on stereotactic coordinates and active contact localization. VC/VS assignment required active contacts or VTA involvement of both the ventral capsule and caudal ventral striatum at the level of the AC.	7 to 9	0 to −1 post. border	−1 to 0
BNST-ALIC	Mai (2004, 2015); Naesström et al. (2021); van der Vlis et al. (2021); Avery et al.(2016); Luyten et al.(2016)	Small nucleus located superior to AC and adjacent to ALIC fibers. The BNST lies dorsally and posteromedially to the NAc, anteromedially to the thalamus, lateral to the fornix, and medial to the ALIC and caudate.	Classification was based on stereotactic coordinates and active contact localization. Active contacts or VTA were required to involve the BNST region and adjacent ALIC fibers.	3 to 6	0 to −2 post. border	0 to 2

Table S8 (suite). Methodology for target definition and classification criteria

Targets	Neuroanatomical References for Target Classification	Anatomical Description and Targeting Considerations of DBS Targets	Criteria for Classification of Stimulation Site and Methodology	Mean coordinates in millimeters (relative to the AC-PC midline)		
				X	Y	Z
ITP	Jiménez et al. (2013)	White matter tract located ventrolateral to the thalamus and posterior to the fornix, connecting the mediodorsal thalamus with orbitofrontal and temporal cortices.	Active contacts or VTA localized within the ITP trajectory between the mediodorsal thalamus and orbitofrontal cortex.	2–3 mm lateral to fornix *	−3 to −5.0 post. border	0 to −0.5
slMFB	Meyer et al. (2022); Coenen et al. (2022)	White matter pathway located within the ventral tegmental region, anterior to the red nucleus and medial to the anteromedial amSTN, connecting mesencephalic and prefrontal regions.	Active contacts localized within the anatomical trajectory of the slMFB based on stereotactic localization and authors' descriptions.	7.6**	−3.3**	−3.0**

x = Mean MNI coordinates in millimeters, lateral to AC-PC midline; y = Mean MNI coordinates in millimeters, anterior to AC; z = Mean MNI coordinates in millimeters, anterior to AC-PC plan. * Coordinates were originally reported relative to fornix: 2–3 mm lateral to fornix (3.5 mm from 3rd ventricle wall; (approximately 6.5 mm lateral to the AC-PC midline). ** Relative to mid-commissural point (MCP). Stimview XT® and Guide XT® (Boston Scientific, Valencia, California & Brainlab, Munich, Germany).

Table S9. Detailed characteristics of included studies

Authors	Year	Study Design	*n*	Follow-up (months, mean)	Hemisphere	DBS-target	Anatomical structure stimulated	Depression scale	Anxiety scale	GAF
Abdelnaim et al.	2023	Case series, retrospective analyses	11	9.6 (range 0-12)	Bilateral	BNST	BNST	MADRS	NR	GAF
Abelson et al.	2005	Randomized, double-blind, ON-OFF design. Followed by unblinded stimulation for the open phase	4	12.9 (range 4-23)	Bilateral	ALIC	vALIC	HAM-D	HAM-A	GAF
Aouizerate et al.	2009	Case reports	2	15	Bilateral	Ventral caudate	Ventral caudate	HDRS	NR	NR
Barcia et al.	2019	Randomized, double-blind, sham-controlled study	7	19	Bilateral	Axial striatum (NAc to caudate)	NAc, n=2 Caudate, n=5	HAM-D, BDI	HAM-A	GAF
Beszlej et al.	2019	Case studies, pilot study	2	6 months	Bilateral	ALIC-NAC	vALIC/ ALIC-NAC	HDRS	HARS	GAF
Chabardès et al.	2020	Prospective, observational, monocentric, open-label cohort (prospectively follow-up of 19 patients included in STOC study, Mallet et al. 2008)	19	24	Bilateral	amSTN (non-motor STN)	amSTN (non-motor STN)	NR	NR	GAF
Coenen et al.	2017	Case series, retrospective analyses	2	12	Bilateral	slMFB	slMFB	NR	NR	NR
Denys et al.	2010	Randomized, double-blind, sham-controlled, crossover study, followed by open-label	16	21	Bilateral	NAc	vALIC	HAM-D	HAM-A	NR
Denys et al.	2020	Prospective, multicenter, open label clinical cohort	70	12	Bilateral	vALIC	vALIC	HAM-D*†	HAM-A*†	NR
Torres Diaz et al.	2021	Case series, multicenter, open label	14	35.9 (range 5-68)	Bilateral	NAc, VS, I.C.	ALIC-NAc	HAM-D	NR	GAF
Farrand et al.	2018	Case series, open label	7	30.9 (range 8-54)	Bilateral	NAc, n=3 BNST, n=3, BNST/NAc, n=1	NAc, n=3 BNST, n=3 BNST/NAc, n=1	DASS-D*	NR	NR
Fayad et al.	2016	Case reports, open label	6	range 72-112	Bilateral	VC/VS	VC/VS	HAM-D, DASS-D	NR	NR
Gadot et al.	2024	Case series, retrospective analyses	10	6	Bilateral	vALIC	vALIC	NR	NR	NR
Goodman et al.	2010	Randomized, double-blind, sham-controlled pilot study	6	12	Bilateral	VC/VS	VC/VS	HDRS*	NR	NR
Graat et al.	2021	Prospective, multicenter, open label	50	80	Bilateral	vALIC	vALIC	HAM-D	HAM-A	GAF*
Greenberg et al.	2006	Case series, prospective	10	36	Bilateral, n=8 Unilateral,n=2	VC/VS	vALIC/NAc and VC/VS	HDRS	HARS*	GAF
Gupta et al.	2019	Case series	2	41.5	Bilateral	VC/VS	VC/VS ventroposterior ALIC	BDI	NR	NR
Holland et al.	2020	Case series, retrospective analyses	9	54.8 (range 5-130)	Bilateral, n=8 Unilateral n=1	VC/VS	VC/VS	NR	NR	NR
Huff et al.	2010	Randomized, double-blind, sham-controlled, crossover study, followed by open label	10	12	Unilateral	Unilateral right NAc	R. NAc, n=7. R. vALIC-NAc, n=3	HDRS, BDI	HARS, STAI	GAF

* Not included in the analysis due to incomplete data reported for statistical calculation.

† Patients included in the study by Denys et al. (2020) are included in another study from Graat et al. (2021).

Table S9. Detailed characteristics of included studies (continued).

Authors	Year	Study Design	*n*	Follow-up (months, mean)	Hemisphere	DBS-target	Anatomical structure stimulated	Depression scale	Anxiety scale	GAF
Huys et al.	2019	Prospective, monocentric, open-label cohort	20	12	Bilateral	vALIC-NAc	vALIC and/or NAc	BDI	NR	NR
Islam et al.	2015	Case series	8	33.8 (range 12-60)	Bilateral	NAc, n=3 BNST, n=5,	vALIC-NAc, n=3 BNST, n=5,	HAM-D *	NR	NR
Jiménez et al.	2013	Case reports	6	24 (range 12-36)	Bilateral	ITP	ITP	NR	NR	GAF*
Kahn et al.	2021	case series	5	25.5 (266-1434 days)	Bilateral	VC/VS	VC/VS	MADRS*	HAM-A*	NR
Lee et al.	2019	Prospective, open label, pilot study	5	49.3 (range 21-85)	Bilateral	ITP	ITP	HAM-D	NR	NR
Luyten et al.	2016	Randomized, double-blind, sham-controlled, crossover study, followed by open stimulation.	24	77.1 (LFU)	Bilateral	ALIC or BNST	vALIC-ALIC, n=6 BNST, n=15 other (comparable to stimulation in ALIC and/or BNST), n=3	HAM-D*	HAM-A*	GAF*
Maarouf et al.	2016	Case series.	4	range 4 -35	Bilateral	MD/VA nucleus (thalamus)	MD/VA nucleus (thalamus)	BDI	STAI	NR
Mallet et al.	2008	Randomized, double-blind, cross-over ON-OFF, multicenter study.	17	10	Bilateral, n=14 Unilateral, n=2	amSTN	amSTN	MADRS*	BAS*	GAF*
Mallet et al.	2019	Prospective (follow-up of RCT - Mallet 2008)	14	40.9 (16-46)	Bilateral, n=12 Unilateral, n=2	amSTN (non-motor STN)	amSTN (non-motor STN)	MADRS*	HARS*	GAF*
Mar-Barrutia et al.	2022	Prospective, observational study, parallel groups: control group (n=25) and DBS (n=25)	50	68	Bilateral	vALIC-NAc, n=7 BNST, n=19	vALIC-NAc, n=7 BNST, n=19	HDRS	NR	GAF
Menchon et al.	2019	Prospective, open label, multi-center study	30	12	Bilateral	AIC	BNST-ALIC (AIC)	MADRS*	NR	GAF*
Meyer et al.	2022	Case series, open label.	9	24.1 (range 11-36)	Bilateral	slMFB	slMFB	MADRS	NR	GAF
Mosley et al.	2021	Randomized, double-blind, sham-controlled trial, followed by open label.	9	15	Bilateral	BNST	BNST	MADRS*	NR	NR
Naesström et al.	2021	Case series, open-label design, control group, pilot study.	11	12	Bilateral	BNST	BNST - ALIC	MADRS	NR	NR
Park et al.	2019	Case series	4	52.50 (range 42-72)	Bilateral	ALIC, n=2 NAc, n=2	ALIC, n=2 NAc, n=2	NR	NR	NR
Parvaresh-Rizi et al.	2022	Case series, open label.	4	11	Bilateral	vALIC-NAC	vALIC-NAC	NR	HAM-A	NR
Roh et al.	2012	Case series, open label.	4	24	Bilateral	VC/VS	NAc, vALIC-NAc	HAM-D	NR	GAF
Tsai et al.	2012	Pilot study, case series	4	15	Bilateral	VC/VS	VC/VS	HAM-D	NR	GAF
Tyagi et al.	2019	Randomized, double-blind, cross-over trial, followed by an open phase.	6	14 (60 weeks)	Bilateral	amSTN, VC/VS	amSTN, vALIC	MADRS*	NR	NR

* Not included in the analysis due to incomplete data reported for statistical calculation.
† Patients included in the study by Denys et al. (2020) are included in another study from Graat et al. (2021).

Table S9. Detailed characteristics of included studies (continued).

Authors	Year	Study Design	*n*	Follow-up (months, mean)	Hemisphere	DBS-target	Anatomical structure stimulated	Depression scale	Anxiety scale	GAF
van den Munckhof et al.	2013	Long term follow-up of Denys 2010.	15	36 (range 11-52)	Bilateral	vALIC, n=9 vALIC-NAC, n=6	vALIC, n=9 vALIC-NAC, n=6	NR	NR	NR
van der Vlis et al.	2021	Case series, retrospective analyses	8	26.25 (10-74)	Bilateral	VC/VS	VC/VS (subpart of the ALIC)	BDI	STAI	NR
Welter et al.	2020	Randomized, double-blind, cross-over trial, followed by an open phase.	8	22	Bilateral	amSTN, NAc, Caudate nucleus	amSTN, NAc, Caudate nucleus	MADRS*	NR	NR
Winter et al.	2021	Case series, prospective, observational study	6	78 (48-98)	Bilateral	BNST-ALIC	ALIC	BDI	NR	NR
Xiong et al.	2023	Cohort study (non controlled)	13	range 12-84	Bilateral	ALIC-NAc	vALIC-NAc	HAM-D	HAM-A	NR

* Not included in the analysis due to incomplete data reported for statistical calculation.

† Patients included in the study by Denys et al. (2020) are included in another study from Graat et al. (2021).

Not reported (NR)

Table S10. Summary of studies reporting stereotactic coordinates, anatomical sites of stimulation, stimulation parameters and DBS-lead models

Authors	Side	DBS Target (reported)	Anatomical structure stimulated	Indirect targeting anatomic coordinates (mean in millimeters; (standard deviation))			Anatomical site of active stimulation - location of active contacts for stimulation (cathode)	Ampli. (mean)	PW (us)	Freq. (Hz)	Electrode model
				X (lateral to midline)	Y	Z					
Abdelnaim et al. 2023	Bilateral	BNST	BNST	NR	NR	NR	Active contacts located in BNST as defined by iPlanNet 3.0 (Brainlab, Munich, Germany).	5.1 V ±0.7	82 ±27.4	147 ±19.5	Medtronic 3387, 3391, 3301
Abelson et al. 2005	Bilateral	ALIC	vALIC-ALIC	Right side: 12.1 Left side: 13.1	Right side: 7.85 A to AC (posterior border) Left side: 7.05 A to AC (posterior border)	Right side: -2.0 to AC-PC Left side: -2.35 to AC-PC	Electrode tip (contact 0) was targeted at the base of vALIc. In one responder, the active contact was the lower contact (0–) located in the vALIC. In second responder, active contacts were the lower to middle contacts (0–, 1–, 2–), located in the vALIC/ALIC.	7.4 V ±2.88	172.5 ±75 range: 50-110	135 ±10 range: 130-150	Medtronic 3387
Aouizerate et al. 2009	Bilateral	Ventral caudate/NAc	Ventral caudate/NAc	Right side: 8.9 Left side: 7.6	Right side: 36.5 A to PC Left side: 31.4 A to PC	Right side: -3.0 to AC-PC Left side: -1.7 to AC-PC	In one patient, contacts 0–, 2–, and 3– were active, reaching the upper border of the NAc and the ventral caudate (limbic and associative territories). In the second patient, stimulation occurred at contact 0–, localized in the caudate nucleus (limbic part) without reaching the NAc.	3 V	105	130	Medtronic 3387
Coenen et al. 2017	Bilateral	slMFB	slMFB	7.6 to MCP*	-3.3 (P) to MCP*	-3.0 to MCP*	Active contacts 2–, 3 (upper), in central portion of the slMFB.	3.1 mA	60	130	Medtronic 3389
Barcia et al. 2019	Bilateral	Axial stritum (NAc to caudate).	NAc, n=2 Caudate, n=5	6.5 L to midline	2.5 A to AC (anterior border)	-4.5 to AC-PC	In 5 patients, the most effective contacts were the upper ones (contacts 2– or 3–) located in the caudate. In one patient, the best contact was the lower one (0–) in the NAc. In another patient, the best effects were observed either with the lower contact (0–) in the NAc or the middle contact (2–) in the caudate. Monopolar cathodic stimulation.	4.5 V	60	130	Medtronic 3391
Beszlej et al. 2019	Bilateral	ALIC-NAC	vALIC/ ALIC-NAC	NR	NR	NR	NR	4 V	105	130	NR
Chabardès et al. 2020	Bilateral	amSTN	amSTN	motor STN: 11-12 L to midline amSTN: 1.0 mm medial to motor STN.	motor STN: 7/12 AC-PC length amSTN: 2.0 mm A to motor STN	-3.0 to MCP plan	All active contacts located in anterior (non-motor) STN; monopolar stimulation.	2 V	NR	NR	Medtronic 3387
Denys et al. 2010	Bilateral	NAc	vALIC	7.0 L to midline	3.0 A to AC (anterior border)	-4.0 to MCP	Upper contacts (2– or 3–) located in NAcc at the junction with vALIC and BNST; stimulation occurred in the vALIC. Bipolar stimulation.	4.3 V range: (3.5-5)	90	130	Medtronic 3389
Denys et al. 2020	Bilateral	vALIC	vALIC	7.0 L to midline	3.0 A to AC (anterior border)	-4.0 to MCP	In participants 1–28, active contacts 2– or 3– were located in the white matter of the vALIC; in participants 29–70, active contacts 1– or 2– were located in vALIC.	Patients 1-28: 4.3V range: 3.5-5	Patients 1-28: 90	Patients 1-28: 130	Medtronic 3389

Model DBS leads dimensions: **3387**: 1.5mm tip lenght, 4 x 1.5mm contacts, 3 x 1.5mm inter contact, 10.5mm contact span excluding tip. **3389**: 1.5mm tip length, 4 x 1.5mm contacts, 3 x 0.5mm inter contact, 7.5mm contact span excluding tip. **3391**: 1.5mm tip length, 4 x 3mm contacts, 3 x 3mm inter contact, 21mm contact span excluding tip. Boston Scientific Vercise **2201** (octopolar): 1.1mm tip length, 8 x 1.5mm contacts,7 x 0.5mm inter contact, 15.5mm span including tip length. Boston Scientific Vercise Cartesia directional **2202** (octopolar): Distal tip is contact 0, 4 levels x 1.5mm contacts, 3 x 0.5mm inter level, 7.5mm span. St Judes ANS 0.5 inter contact.

Table S10. Summary of studies reporting stereotactic coordinates, anatomical sites of stimulation, stimulation parameters and DBS-lead models (continued).

**Authors**	**Side**	**DBS Target** (reported)	**Anatomical structure stimulated**	**Indirect targeting anatomic coordinates** (mean in millimeters; (standard deviation))			**Anatomical site of active stimulation - location of active contacts for therapeutic stimulation (cathode)**	**Ampli.** (mean)	**PW** (us)	**Freq.** (Hz)	**Electrode model**
				X	Y	Z					
Farrand et al. 2018	Bilateral	BNST	NAc, n=3 BNST, n=3, BNST/NAc, n=1	NR	NR	NR	In 3 patients, the target was bilateral NAc (tip implanted 2-4 mm beyond target. in 3 patients, bilateral BNST: 1 mm posterior to AC, 2-3 mm below AC level, 4 mm lateral to midline; trajectory through ALIC and AC–ALIC junction.; and in one patient, left NAc and right BNST.	NR	NR	NR	n=5, Medtronic3389 n=2, St-Jude ANS
Fayad et al. 2016	Bilateral	VC/VS	VC/VS	*Coordinates of the center of active contact relative to the midcommissural point* (MCP) (mean in mm; SD).			Long term settings: In 4 patients, the two lower contacts (0- and/or 1-) are the active contacts. In 2 patients, the middle contacts (1-,2-) are the active contacts. Monopolar and multi-monopolar.	5.3 V range: 4.8 to 5	180 range: 150-210	132.5 range: 130-135	Medtronic 3887
				Right side: 8.9 (2.10) L to MCP Left side: 11.0 (2.57) L to MCP	Right side: 15.3 (2.47) A to MCP Left side: 16.1 (1.90) A to MCP	Right side: 0.6 (4.42) to MCP Left side: 0.9 (5.33) to MCP					
				4 responders Right side: 8.5 (2.49) L to MCP Left side: 10.0 (2.59) L to MCP	*4 responders* Right side: 15.6 (2.43) A to MCP Left side *: 15.6 (2.21) A to MCP	*4 responders* Right side: -0.6 (2.66) to MCP Left side: -0.5 (4.16) to MCP	Long term settings amongst responders: in 3 responders, the active contacts are the middle contact (1-) and in one responder, the middle contacts (1-) and (2-). Monopolar and multi-monopolar.	5.3 V range: 4.8 to 5	180 range: 150-210	132.5 range: 130-135	Medtronic 3887
Gadot et al. 2024	Bilateral	vALIC	vALIC	NR	NR	NR	Two lower contacts (0- or 1-) in vALIC. Monopolar stimulation.	4.9 mA ±0.7	NR	NR	Medtronic 3387
Goodman et al. 2010	Bilateral	VC/VS	VC/VS	*Coordinates of the centers of active contacts relative to the midcommissural point* (mean in mm; SD).			In 4 patients, the active contacts are the two lower contacts (0- and/or 1-). In one patient, the active contact is the middle one (1-). In one patient, the active contact is (2-).	5.5 V	165	133	Medtronic 3887
				4 responders Right side: 8.5 (2.49) L to MCP Left side: 10.0 (2.59) L to MCP	*4 responders* Right side: 15.6 (2.43) A to MCP Left side *: 15.6 (2.21) A to MCP	*4 responders* Right side: -0.6 (2.66) to MCP Left side: -0.5 (4.16) to MCP	In 4 responders, the active contacts are the two lower contacts (0- and/or 1-). Patients reported in Fayal et al. (2016) for long term follow-up.	5.5 V	165	133	
Graat et al. 2021	Bilateral	vALIC	vALIC	7.0 L to midline	3.0 A to AC (anterior border)	-4.0 to AC-PC plan	In participants 1-28 the active contacts 2- or 3- were located in the white matter of the vALIC. In participants 29-50 the active contacts 1- or 2- located in vALIC.	4.8 V ±1	90	130	Medtronic 3389

Table S10. Summary of studies reporting stereotactic coordinates, anatomical sites of stimulation, stimulation parameters and DBS-lead models (continued).

Authors	Side	DBS Target (re-ported)	Anatomical structure stimulated	Indirect targeting anatomic coordinates (mean in millimeters; (standard deviation))			Anatomical site of active stimulation - location of active contacts for therapeutic stimulation (cathode)	Ampli. (mean)	PW (us)	Freq. (Hz)	Electrode model
				X	Y	Z					
Greenberg et al. 2006	Bil, n=8<br><br>Uni. n=2	VC/VS	vALIC/NAc and VC/VS	***vALIC-NAc*, n=3**<br>Right: 10 L to mid.<br>Left: 9.7 L to mid.<br><br>***VC/VS*, n=5**<br>Right: 7.5 L to mid.<br>Left: 8.3 L to mid.<br><br>***P. VC/VS* n=1**<br>posterior/caudal NAc/BNST)<br>Right: 5.5 L to mid.<br>Left: 7.1 L to mid.	***vALIC-NAc* , n=3**<br>Right: 6.2 AC<br>Left: 5.5 AC<br><br>***VC/VS, n=5***<br>Right: 3.0 AC<br>Left: 2.5 AC<br><br>***P. VC/VS, n=1***<br>(posterior/caudal NAc/BNST)<br>Right: 0.4 P to AC<br>Left: 0.2 P to AC<br>(1-2 mm P to AC)	***vALIC-NAc*, n=3**<br>Right: -3.0 to AC-PC<br>Left: -3.7 to AC-PC<br><br>***VC/VS*, n=5**<br>Right: -3.0 to AC-PC<br>Left: -3.8 to AC-PC<br><br>***P. VC/VS*, n=1**<br>posterior/caudal NAc/BNST)<br>Right: -3.9 to AC-PC<br>Left: -3.7 to AC-PC	In 7 patients, the active contacts were the two lower contacts (0 - and/or 1-) in NAc/junction vALIC. In 3 patients, the active contacts were the lower and the middle ones (0-, 1-, 2-), stimulating the caudal NAc, vALIC and dorsal ALIC.<br>Monopolar and bipolar stimulation. Stimulation in posterior targets (VC/VS) required lower amplitude and pulse width to reach therapeutical benefits.	range:<br>2 -15mA<br><br>range:<br>2-8 V<br><br>vALIC-NAC:<br>8.4V<br><br>VC/VS:<br>7.4 V<br><br>Caudal VC/VS:<br>4.9 V	range:<br>90-450<br><br>vALIC-NAC:<br>310<br><br>VC/VS:<br>235<br><br>Caudal VC/VS:<br>186	100-130	Medtronic 3387
Gupta et al. 2019	Bilateral	VC/VS	VC/VS ventroposterior ALIC	5.0 - 6.0 L to midline	0-1 P to AC	slightly below AC-PC plan.	The stimulation occurred in the ventroposterior part of ventral capsule below vALIC according to Stereotactic Atlas.	NR	NR	NR	Medtronic 3387
Holland et al. 2020	Bil. n=8<br>Uni. n=1	VC/VS	VC/VS	NR	NR	NR	In 8 patients, the active contacts were the two lower contacts (0- and/or 1-) located in the VS. In one patient, unilateral stimulation administered through the contacts in ventral capsule. Monopolar and bipolar stimulation.	5.1 V ±1<br>range:<br>2.5-8	170<br>±52.5	125.5<br>±18	Med-tronic3391
Huff et al. 2010	Uni.	Uni.<br>right NAc	R. NAc, n=7.<br>R. vALIC-NAc, n=3	*Coordinates relative to midline AC-PC* (mean in mm; SD). One patient responder ≥ 35 % Y-BOCS. Coordinates for contacts from distal to dorsal: 0,1,2,3.			In 7 patients, the two lower contacts (0- and 1) are active and placed in the right NAc. In 3 patients, the actives contacts (1-, 2-, 3-) are located in right vALIC. Monopolar stim.	5.5 V<br>range<br>4.5-6.5	90	145	Medtronic 3387
				0 — 7.2 (0.5) L<br>Responder:<br>7.3 L	-0.2 (1.6) to AC<br>(anterior border)<br>Responder:<br>-1.9 A to AC	-4.1 (1.2)<br>to AC-PC<br>Responder:<br>-3.6 to AC-PC.					
				1 — 8.8 (0.5) L<br>Responder:<br>8.9 L	1.3 (1.7) to AC<br>(anterior border)<br>Responder:<br>0.1 A to AC.	-2.1 (1.1)<br>to AC-PC<br>Responder:<br>-1.4 to AC-PC.					
				2 — 10.4 (0.6) L<br>Responder:<br>10.2 L	2.9 (1.8) to AC<br>(anterior border)<br>Responder:<br>2.0 A to AC.	-0.1 (1.0)<br>to AC-PC.<br>Responder:<br>0.70 to AC-PC					
				3 — 11.9 (0.7) L<br>Responder:<br>10.2 L	4.4 (2.0) to AC<br>(anterior border)<br>Responder:<br>3.9 A to AC.	1.8 (0.9)<br>to AC-PC<br>Responder:<br>2.7 to AC-PC.					

Table S10. Summary of studies reporting stereotactic coordinates, anatomical sites of stimulation, stimulation parameters and DBS-lead model (continued).

**Authors**	**Side**	**DBS Target**	**Anatomical structure stimulated**	**Indirect targeting anatomic coordinates** (mean in millimeters; (standard deviation))			**Anatomical site of active stimulation - location of active contacts for therapeutic stimulation (cathode)**	**Ampli.** (mean)	**PW** (us)	**Freq.** (Hz)	**Electrode model**
				X	Y	Z					
Huys et al. 2019	Bilateral	vALIC-NAc	vALIC and/or NAc	NR	NR	NR	n=9, two upper contacts (2-, 3-) in vALIC. n=6, middle contacts (1-, 2-) in vALIC-NAc. n=5, two lower contacts (0-, 1-) in NAc.	4.0 V ±1.1 range: 3-6.5	134 ±12.7 range: 120-180	115.2 ±48 range: 90-210	Medtronic 3389 3387
Islam et al. 2015	Bilateral	NAc, n=3 BNST, n=5	vALIC-NAc, n=3 BNST,n=5	NR	NR	NR	In 3 patients, the active contacts are the two middle contacts (1-, -2-) located in the NAc. In one patient, the active contact is the contact 2-. In 4 patients, the active contact is the lower contact (0-) located in the BNST. Monopolar and bipolar stimulation.	4.81 V ±0.6	165 ±62.1 NAc: 92 BNST: 210	136.25 ±17.7	Medtronic 3389
Jiménez et al. 2013	Bilateral	ITP	ITP	2.0 to 3.0 L to fornix (3.5 L to 3rd ventricle wall)	3.0 to 5.0 P to AC	0	Middle contacts (1-, 2-) closest to ITP. Bipolar stimulation.	range: 3-5 V	450	130	Medtronic 3387
Kahn et al. 2021	Bilateral	VC/VS	VC/VS	7.0 to 10.0 L to midline	0 to 5.0 A to AC	-1.0 to -5.0 to the inferior border of AC on the AC-PC.	The active contacts were the two lower contacts (0- or 1-) located in ALIC at the junction with AC. The 0–1 intercontact space lies at the AC–ALIC junction. Results indicate that the closer the active contact's VTA is to this junction, the better the clinical outcomes. Monopolar and bipolar stimulation.	4.6 V 5.7 mA	133	130	Medtronic 3391
Lee et al. 2019	Bilateral	ITP	ITP	6.5 L to midline	3.0 P to posterior margin of AC	-0.5 to AC-PC	Middle contact (1-) in 3 patients. Middle contact (2-) in 2 patients.	6.8 V range: 5.0-8.5	90.4	130	n=3, Medtronic 3387 n=2, 6143ANS
Luyten et al. 2016	Bilateral	ALIC or BNST	vALIC/ALIC n=6 BNST, n=15 Other comparable to ALIC and/or BNST stimulation. n=3	NR	NR	NR	In most patients, the active contacts were the two lower contacts (0- and/or 1-). Results are reported by site of stimulation by the authors. Monopolar and bipolar stimulation.	6.3 V Range: 3.0-10.5 BNST: 6.6 V ±2.1 ALIC: 6.7 V ±2.3	270 range: 90-450 BNST: 210 range 120-450 ALIC: 345 range: 240-450	115 range: 85-130	Medtronic 3887 3387 3391

Table S10. Summary of studies reporting stereotactic coordinates, anatomical sites of stimulation, stimulation parameters and DBS-lead models (continued).

**Authors**	**Side**	**DBS Target**	**Anatomical structure stimulated**	**Indirect targeting anatomic coordinates** (mean in millimeters; (standard deviation))				**Anatomical site of active stimulation - location of active contacts for therapeutic stimulation (cathode)**	**Ampli.** (mean)	**PW** (us)	**Freq.** (Hz)	**Electrode model**
					X	Y	Z					
Maarouf et al. 2016	Bilateral	MD/VA nuclei thalamus	MD/VA nuclei thalamus	*Coordinates (center of active contacts) relative to midcommissural AC-PC (MCP).* (mean in mm; SD). Coordinates for contacts from distal to dorsal: 0,1,2,3.				In 3 patients, the active contacts are the lower and the two middle contacts (0-,1-,2-) located in the MD nucleus. In one patient, the active contacts are the two upper contacts (2-,3-) located in the MD and VA nuclei.	3.1 V range: 2.5-3.5	105 range: 90-120	130	Medtronic 3387 3389
				0	4.1 (1.0) L to midline	20.0 (1.4) P to AC	3.8 (1.0) to MCP					
				1	4.9 (0.8) L to midline	18.2 (1.2) P to AC	5.1 (1.6) to MCP					
				2	5.7 (0.7) L to midline	16.1 (1.2) P to AC	6.4 (1.3) to MCP					
				3	6.6 (0.6) L to midline	14.2 (1.3) P to AC	7.7 (1.7) to MCP					
Mallet et al. 2008	Bilateral n=14 Uni. n=2	amSTN	amSTN		motor STN: 11-12 L to midline amSTN: 1 mm medial to the motor STN	motor STN: 7/12 AC-PC length P to AC. amSTN: 2 mm A to the motor STN	-2.2 to AC (post. border)	For most active contacts, the stimulation reached the amSTN (limbic territory).	2.1 V ±0.8	60	130	Medtronic 3389
Mallet et al. 2019	Bilater-aln=12 Uni. n=2	amSTN	amSTN		motor STN: 11-12 L to midline amSTN: 1 mm medial to motor STN	motor STN: 7/12 AC-PC length P to AC. amSTN: 2 mm A to motor STN	-2.2 to AC (post. border)	In the 14 patients, the stimulating contacts are localised in the am-STN (non-motor STN) part, which correspond to the associative-limbic part of the SNT.	2.1 V ±0.8	60	130	Medtronic 3389
Mar-Barrutia et al. 2022	Bilateral	vALIC-NAc, n=7 BNST, n=19	vALIC-NAc, n=7 BNST, n=19		NAc: 6.0 L to midline. BNST: 5.0 to 6.0 L to posterior border of AC.	2.0 A to AC	-4.0 to AC	Lower contacts in white matter in 14 patients. Two lower contacts (0-,1-) in BNST in 11 patients.	3.7 mA range: 3-5.5	193 range: 90-210	130	NAc: Medtronic 3389 BNST: Medtronic 3391
Menchon et al.2019	Bilateral	AIC	BNST-ALIC (AIC)		NR	NR	NR	In responders at 12 months, the active contacts were located bilaterally within AIC in 6 patients or in the BNST in 6 patients. Amongst nonresponders, 4 patients had active contacts located bilaterally within ALIC or 3 patients were not receiving active stimulation. 56 % of the active contacts were located in the ALIC; 27% of the active contacts were located in the BNST.	4.7 V ±1.8	221 ±63	130 ±3	Medtronic 3391

Table S10. Summary of studies reporting stereotactic coordinates, anatomical sites of stimulation, stimulation parameters and DBS-lead models (continued).

**Authors**	**Side**	**DBS Target**	**Anatomical structure stimulated**	**Indirect targeting anatomic coordinates**<br>(mean in millimeters; (standard deviation))			**Anatomical site of active stimulation - location of active contacts for therapeutic stimulation (cathode)**	**Ampli.**<br>(mean)	**PW**<br>(us)	**Freq.**<br>(Hz)	**Electrode model**
				X	Y	Z					
Meyer<br>et al. 2022	Bilateral	slMFB	slMFB	NR	NR	NR	All electrodes are located in the ventral tegmentum, anterior to the red nucleus and medial to the STN. Monopolar and bipolar.	range:<br>1.4-5.5<br>mA	range:<br>30-150	130	BSC<br>Cartesia<br>2202
Mosley<br>et al. 2021	Bilateral	BNST	BNST	NR	NR	NR	The active contacts selected were located in the superolateral region of the hypothalamus, with stimulation in the BNST, adjacent to the posterior border of the Nacc and inferomedial to the ventral pallidum. Stimulated VTA was estimated in each hemisphere for each patient to confirm the localisation and the intersection of this VAT for each structure. For most patients, the actives contacts were the middle 1- and/or 2-.	4.6 V<br>±0.5<br>range:<br>3.5-5.6	93.8<br>± 10.3<br>range:<br>90-120	130	Medtronic<br>3389
Naesstrom<br>et al. 2021	Bilateral	BNST	BNST-ALIC	NR	NR	NR	Most of the active contacts were the middle contacts (1- and 2-) located in the BNST and the ALIC.	4.2 V<br>±0.5<br>range:<br>3.2-4.9	87<br>± 28<br>range:<br>60-150	130	Medtronic<br>3387
Park<br>et al. 2019	Bilateral	ALIC, n=2<br>NAc, n=2	ALIC, n=2<br>NAc, n=2	NR	NR	NR	NR	ALIC:<br>2.8 ±1.8<br>NAc:<br>2.3 ±1.1	60	130	Medtronic<br>3387
Parvaresh-<br>Rizi<br>et al. 2022	Bilateral	vALIC-NAC	vALIC-NAC	NR	NR	NR	NR	NR	NR	NR	Medtronic<br>3391
Roh<br>et al. 2012	Bilateral	VC/VS	NAc, n = 1<br>vALIC-<br>NAc,n =3	8.0 L to midline	4.0 A to posterior border of AC.	-2.0<br>to AC-PC	In one patient, the active contact is the lower contact (0-) located in the NAc. In 3 patients, the active contacts are the two lower contacts (0- and 1-) located in NAc extending into I.C.	3.9 V<br>±1.31<br>range:<br>2-5	165<br>± 79.4<br>range:<br>90-127	120<br>± 20<br>range:<br>90-130	Medtronic<br>3387
Torres<br>Diaz<br>et al. 2021	Bilateral	NAc, VS,<br>I.C.	ALIC-NAc	NAC:<br>5.0 L to midline<br>VS:<br>10.0 L to midline<br>I.C.:<br>20.0 L to midline	NAC:<br>3.0 A to AC<br>VS:<br>5.0 A to AC<br>I.C:<br>10.0 A to AC	NAC:<br>-4.0 to AC-PC<br>VS:<br>-0.0 to AC-PC<br>I.C.:<br>5.0 to AC-PC	In 7 patients, the stimulation is delivered through the lower contact (0-) located in the NAc. In one patient, the active contact is the lower one located in the ventral caudate nucleus (VS). In 2 patients, the active contact is the upper one (3-) corresponding to the I.C.	range:<br>3-5 mA	90	130	BSC<br>Vercise<br>2201<br>Medtronic<br>3387<br>3391
Tsai<br>et al. 2012	Bilateral	VC/VS	VC/VS	*Coordinates of active contacts set as the cathode*<br>(mean in millimeters; standard deviation (SD))<br>Coordinates for contacts from distal to dorsal: 0,1,2,3.			In 2 patients, the lower contact (0) is the active contact located into the vALIC and VS. In one patient, the active contact is the first middle contact (1-) located into the VS and vALIC. In one patient, the active contact is the second middle contact (2-) at ventral capsule.	3.6 V<br>range:<br>2-6	210	130	Medtronic<br>3387
				0 Right side:<br>7.5 (2.1) L<br>to midline<br>Left side:<br>7.9 (1.1) L<br>to midline	Right side:<br>15.5 (3.9) A<br>to MCP.<br>Left side:<br>15.8 (3.5) A<br>to MCP.	Right side:<br>-3.7 (0.1)<br>to AC-PC<br>Left side:<br>-3.6 (1.8)<br>to AC-PC					

Table S10. Summary of studies reporting stereotactic coordinates, anatomical sites of stimulation, stimulation parameters and DBS-lead models (continued).

**Authors**	**Side**	**DBS Target**	**Anatomical structure stimulated**	**Indirect targeting anatomic coordinates (mean in millimeters; (standard deviation)**				**Anatomical site of active stimulation - location of active contacts for therapeutic stimulation (cathode)**	**Ampli.** (mean)	**PW** (us)	**Freq.** (Hz)	**Electrode model**
					X	Y	Z					
Tsai et al. 2012 (suite)	Bilateral	VC/VS	VC/VS	1	Right side:<br>10.2 L<br>to midline<br>Left side:<br>7.6 L<br>to midline	Right side:<br>16.8 A<br>to MCP.<br>Left side:<br>16.0 A<br>to MCP.	Right side:<br>-1.2<br>to AC-PC<br>Left side:<br>-0.6<br>to AC-PC					
				2	Right side:<br>9.9 L<br>to midline<br>Left side:<br>9.6 L<br>to midline	Right side:<br>17.3 A<br>to MCP.<br>Left side:<br>17.5 A<br>to MCP	Right side:<br>-0.2<br>to AC-PC<br>Left side:<br>-0.1<br>to AC-PC					
				3	The proximal contact is not a stimulating active contact.	The proximal contact is not a stimulating active contact.	The proximal contact is not a stimulating active contact.					
Tyagi et al. 2019	Bilateral	amSTN, VC/VS	amSTN, vALIC		8.0 L<br>to midline	4.0 A to the A.C	-6.0<br>to AC-PC	VC-VS:<br>Contacts 0 and 1 located in the NAc shell and core respectively. Contacts 2, 3 in the vALIC. The most effective contacts (2-, 3-) were located in the vALIC. The average VTA was centered in the white matter in vALIC.<br>amSTN:<br>active contacts were the two lower contacts (0- or 1-).	amSTN:<br>1.9 V<br>±0.5<br>vALIC:<br>6.4 V<br>±0.6<br>range:<br>0-8	NR	NR	amSTN:<br>Medtronic3389<br>vALIC:<br>Medtronic 3387
van den Munckhof et al. 2013	Bilateral	vALIC, N=9<br>vALIC-NAC, N=6	vALIC, N=9<br>vALIC-NAC, N=6	*	7.0 L<br>to midline	3.0 A to AC.<br>(posterior border)	-4.0<br>to AC-PC	In 9 patients, the centers of the stimulation were located in vALIC, more lateral, anterior, and dorsal to the NAc, or at the border between vALIC and NAc or Cd. In 6 patients, the centers of the stimulation were located otherwise. Monopolar stimulation.	4.7 V<br>±0.6<br>range:<br>3.5-5.6	103<br>±24<br>range:<br>90-150	145<br>±0.25<br>range:<br>130-185	Medtronic 3389
				**	Left:<br>10.8 (1.3) L<br>to midline<br>Right:<br>11.1 (1.0) L<br>to midline	Left:<br>4.4 (1.0) A to AC<br>(posterior border)<br>Right:<br>3.9 (1.2) A to AC<br>(posterior border)	Left:<br>2.4 (1.3)<br>to AC-PC<br>Right:<br>2.0 (1.8)<br>to AC-PC					
				***	11 L<br>to midline	4 A to the anterior border of AC.	3<br>to AC-PC					
van der Vlis et al. 2021	Bilateral	VC/VS	VC/VS (subpart of the ALIC)		6.4 L	13.56 A<br>to MCP	-2.5<br>to MCP	In 4 patients, the active contacts were the lower contacts (0-). In 3 patients, the active contacts are the middle contacts (1-) in one patient; (2-) in 2 patients. In one patient, the active contacts were the upper ones (3-).	5.8V	90	130	Medtronic 3387

** Stereotactic target coordinates for electrode tip; ** Stereotactic coordinates of the centers of stimulation in OCD patients; *** Recommended coordinates for the center of stimulation (Morel A. 2007)*

Table S10. Summary of studies reporting stereotactic coordinates, anatomical sites of stimulation, stimulation parameters and DBS-lead models (continued).

Authors	Side	DBS Target	Anatomical structure stimulated	Indirect targeting anatomic coordinates (mean in millimeters; (standard deviation)			Anatomical site of active stimulation - location of active contacts for therapeutic stimulation (cathode)	Ampli. (mean)	PW (us)	Freq. (Hz)	Electrode model
				X	Y	Z					
Welter et al. 2020	Bilateral	amSTN, NAc, Caudate	amSTN, NAc, Caudate	NR	NR	NR	NR	NR	NR	NR	NR
Winter et al. 2021	Bilateral	BNST-ALIC	ALIC	2.0 ± 1.0 L to midline	-2.0 ± 1.0 to AC	0 to AC-PC	At the last follow-up, all patients are stimulated in ALIC. In 4 patients, the middle contact (2-) in AIC is active. In one patient, the upper contact (3-) in AIC is the active contact. In one patient, the active contact (1-) located at the junction BNST/AIC.	4.4 ±2.0 range: 1.1-6.4	197 ±31.1 range: 130-210	143.3 ±31.1 range: 130-210	Medtronic 3387
Xiong et al. 2023	Bilateral	ALIC-NAc	vALIC-NAc	7.0 ± 2.0 L to midline	5.0 ± 2.0 A to AC (posterior border)	-5.0 ± 2.0 to AC-PC	Simultaneously stimulation: dorsal contacts in the NAc (0- and/or 1-) and ventral contacts (2-) in ALIC (vALIC).	NAc: 3.8 V ±1.2 range: 2.8-6<br>ALIC: 5 V ±1.5 range: 3-7	NAc: 209 ±61 range: 120-300<br>ALIC: 207 ±40.7 range: 120-280	NAc: 121.5 ±21.5 range: 60-130<br>ALIC: 121.5 ±21.5 range: 60-130	BSC Vercise 2201; Medtronic 3387 3389

NR, not reported; **A**, anterior; **AC**, anterior commissure; **AIC**, anterior internal capsule; **ALIC**, anterior limb of the internal capsule; **amSTN**, antero-medial subthalamic nucleus; **BNST**, bed nucleus of the stria terminalis; **I.C.**, internal capsule; **ITP**, inferior thalamic peduncle; **L**, lateral; **MCP**, midcommissural point; **NAc**, nucleus accumbens; **NAcc**, nucleus accumbens core; **PC**, posterior commissure; **S**, superior; **slMFB**, superolateral middle forebrain; **vALIC**, ventral anterior limb of the internal capsule; **VC**, ventral capsule; **VS**, ventral striatum

Table S11. PRISMA 2020 Main Checklist

Topic	No.	Item	Location where item is reported
**TITLE**			
**Title**	1	Identify the report as a systematic review.	YES. Identify in the title
**ABSTRACT**			
**Abstract**	2	See the PRISMA 2020 for Abstracts checklist	Abstract: p 1<br>Methods: P. 4
**INTRODUCTION**			
**Rationale**	3	Describe the rationale for the review in the context of existing knowledge.	Introduction: p.2-3.
**Objectives**	4	Provide an explicit statement of the objective(s) or question(s) the review addresses.	Introduction: p.3.
**METHODS**			
**Eligibility criteria**	5	Specify the inclusion and exclusion criteria for the review and how studies were grouped for the syntheses.	Search strategy and selection criteria section: p. 4.
**Information sources**	6	Specify all databases, registers, websites, organisations, reference lists and other sources searched or consulted to identify studies. Specify the date when each source was last searched or consulted.	Search strategy and selection criteria section: p. 3-4; Supplementary Materials Tables S1 and S2.
**Search strategy**	7	Present the full search strategies for all databases, registers and websites, including any filters and limits used.	Search strategy and selection criteria section: p. 4; Supplementary Materials Tables S1 and S2.
**Selection process**	8	Specify the methods used to decide whether a study met the inclusion criteria of the review, including how many reviewers screened each record and each report retrieved, whether they worked independently, and if applicable, details of automation tools used in the process.	Selection process and data collection section: p. 4.
**Data collection process**	9	Specify the methods used to collect data from reports, including how many reviewers collected data from each report, whether they worked independently, any processes for obtaining or confirming data from study investigators, and if applicable, details of automation tools used in the process.	Selection process and data collection section: p. 3-4.

Topic	No.	Item	Location where item is reported
**Data items**	10a	List and define all outcomes for which data were sought. Specify whether all results that were compatible with each outcome domain in each study were sought (e.g. for all measures, time points, analyses), and if not, the methods used to decide which results to collect.	Data items section: Bullet points, p. 4.
	10b	List and define all other variables for which data were sought (e.g. participant and intervention characteristics, funding sources). Describe any assumptions made about any missing or unclear information.	Data items section: Bullet points and Endpoints and sub analysis sections, p. 4.
**Study risk of bias assessment**	11	Specify the methods used to assess risk of bias in the included studies, including details of the tool(s) used, how many reviewers assessed each study and whether they worked independently, and if applicable, details of automation tools used in the process.	Assessment of quality study section: first paragraph - Risk of bias assessment, p. 5.
**Effect measures**	12	Specify for each outcome the effect measure(s) (e.g. risk ratio, mean difference) used in the synthesis or presentation of results.	Endpoints and sub-analysis section; Secondary outcomes, p. 5.
**Synthesis methods**	13a	Describe the processes used to decide which studies were eligible for each synthesis (e.g. tabulating the study intervention characteristics and comparing against the planned groups for each synthesis (item 5)).	Selection process and data collection section, p. 4.
	13b	Describe any methods required to prepare the data for presentation or synthesis, such as handling of missing summary statistics, or data conversions.	Endpoints and sub-analysis section; Secondary outcomes, p. 4.
	13c	Describe any methods used to tabulate or visually display results of individual studies and syntheses.	Statistical analysis section, p. 6.
	13d	Describe any methods used to synthesize results and provide a rationale for the choice(s). If meta-analysis was performed, describe the model(s), method(s) to identify the presence and extent of statistical heterogeneity, and software package(s) used.	Statistical analysis section, page 5; Risk of bias analysis section, p. 10.
	13e	Describe any methods used to explore possible causes of heterogeneity among study results (e.g. subgroup analysis, meta-regression).	Statistical analysis section, p. 5.
	13f	Describe any sensitivity analyses conducted to assess robustness of the synthesized results.	Sensitivity analysis section, p. 16; Supplementary Materials Table S5

Topic	No.	Item	Location where item is reported
**Reporting bias assessment**	14	Describe any methods used to assess risk of bias due to missing results in a synthesis (arising from reporting biases).	Assessment of quality study section, p. 5.
**Certainty assessment**	15	Describe any methods used to assess certainty (or confidence) in the body of evidence for an outcome.	Assessment of quality study section, p. 5; Supplementary Materials, section on Quality of the evidence based on GRADE approach, p. 21-25
**RESULTS**			
**Study selection**	16a	Describe the results of the search and selection process, from the number of records identified in the search to the number of studies included in the review, ideally using a flow diagram.	Results - Study selection: p.7. Figure 1: 2020 Prisma Flow chart diagram, p.8. Supplementary Materials: Tables S1 and S2.
	16b	Cite studies that might appear to meet the inclusion criteria, but which were excluded, and explain why they were excluded.	Results – Study selection, p. 7. Figure 1: 2020 Prisma Flow chart diagram, p.8.
**Study characteristics**	17	Cite each included study and present its characteristics.	Study characteristics sections, p.8-9; Table 1, p.8.
**Risk of bias in studies**	18	Present assessments of risk of bias for each included study.	Detailed assessments of risk of bias are presented in Supplementary Materials, Figure S1.
**Results of individual studies**	19	For all outcomes, present, for each study: (a) summary statistics for each group (where appropriate) and (b) an effect estimate and its precision (e.g. confidence/credible interval), ideally using structured tables or plots.	Meta-analysis results for primary outcome, p.11-14; Figures 2-3; Table 2: Therapeutics effects of DBS on OCD,p. 14.
**Results of syntheses**	20a	For each synthesis, briefly summarise the characteristics and risk of bias among contributing studies.	Meta-analysis results for primary outcome, p.11-14; Figures 2-3; Table 2: Therapeutics effects of DBS on OCD, p.14.; Supplementary Material, Figures S1 and S2.
	20b	Present results of all statistical syntheses conducted. If meta-analysis was done, present for each the summary estimate and its precision (e.g. confidence/credible interval) and measures of statistical heterogeneity. If comparing groups, describe the direction of the effect.	Meta-analysis results for primary outcome, p.11-14; Figures 2-3; Table 2: Therapeutics effects of DBS on OCD, p.14.; Supplementary Material, Tables S4-S7.

Topic	No.	Item	Location where item is reported
	20c	Present results of all investigations of possible causes of heterogeneity among study results.	Meta-regression analyses, p.16; Supplementary Table S7; Supplementary Materials, section on Quality of the evidence based on GRADE approach, p. 21-25
	20d	Present results of all sensitivity analyses conducted to assess the robustness of the synthesized results.	Sensitivity analyses, p.16; Table 2; Supplementary Materials, Tables S4-S7; Figure S2.
**Reporting biases**	21	Present assessments of risk of bias due to missing results (arising from reporting biases) for each synthesis assessed.	Meta-regression analyses, p.16; Supplementary Table S7; Figure S2; Supplementary Materials, section on Quality of the evidence based on GRADE approach, p. 21-25
**Certainty of evidence**	22	Present assessments of certainty (or confidence) in the body of evidence for each outcome assessed.	Quality assessment, p.16; Supplementary Material, section on Quality of the evidence based on GRADE approach, p. 21-25; Figures S4 and S5.
**DISCUSSION**			
**Discussion**	23a	Provide a general interpretation of the results in the context of other evidence.	Discussion section – Main findings, p.17.
	23b	Discuss any limitations of the evidence included in the review.	Discussion section – Limitations, p.18-19.
	23c	Discuss any limitations of the review processes used.	Discussion section – Limitations, p.18-19.
	23d	Discuss implications of the results for practice, policy, and future research.	Discussion section – Implications, p.19-20.
**OTHER INFORMATION**			
**Registration and protocol**	24a	Provide registration information for the review, including register name and registration number, or state that the review was not registered.	The Review was not registered
	24b	Indicate where the review protocol can be accessed, or state that a protocol was not prepared.	The Review was not registered

Topic	No.	Item	Location where item is reported
	24c	Describe and explain any amendments to information provided at registration or in the protocol.	The Review was not registered
**Support**	25	Describe sources of financial or non-financial support for the review, and the role of the funders or sponsors in the review.	No funding, p. 21.
**Competing interests**	26	Declare any competing interests of review authors.	Statement: Conflict of interest, p.21.
**Availability of data, code and other materials**	27	Report which of the following are publicly available and where they can be found: template data collection forms; data extracted from included studies; data used for all analyses; analytic code; any other materials used in the review.	Available upon request to the authors: template data collection forms, data extracted from included studies, data used for analyses, statistical analyses.

PRIMSA Abstract Checklist

Topic	No.	Item	Reported?
**TITLE**			
**Title**	1	Identify the report as a systematic review.	Yes
**BACKGROUND**			
**Objectives**	2	Provide an explicit statement of the main objective(s) or question(s) the review addresses.	Yes
**METHODS**			
**Eligibility criteria**	3	Specify the inclusion and exclusion criteria for the review.	Yes
**Information sources**	4	Specify the information sources (e.g. databases, registers) used to identify studies and the date when each was last searched.	Yes
**Risk of bias**	5	Specify the methods used to assess risk of bias in the included studies.	Yes
**Synthesis of results**	6	Specify the methods used to present and synthesize results.	Yes
**RESULTS**			
**Included studies**	7	Give the total number of included studies and participants and summarise relevant characteristics of studies.	Yes

Topic	No.	Item	Reported?
**Synthesis of results**	8	Present results for main outcomes, preferably indicating the number of included studies and participants for each. If meta-analysis was done, report the summary estimate and confidence/credible interval. If comparing groups, indicate the direction of the effect (i.e. which group is favoured).	Yes
**DISCUSSION**			
**Limitations of evidence**	9	Provide a brief summary of the limitations of the evidence included in the review (e.g. study risk of bias, inconsistency and imprecision).	Yes
**Interpretation**	10	Provide a general interpretation of the results and important implications.	Yes
**OTHER**			
**Funding**	11	Specify the primary source of funding for the review.	Yes
**Registration**	12	Provide the register name and registration number.	No

From: Page MJ, McKenzie JE, Bossuyt PM, Boutron I, Hoffmann TC, Mulrow CD, et al. The PRISMA 2020 statement: an updated guideline for reporting systematic reviews. MetaArXiv. 2020, September 14. DOI: 10.31222/osf.io/v7gm2. For more information, visit: www.prisma-statement.org

Figure S1. Risk of bias assessment for randomized controlled trials using the Cochrane RoB 2 tool. (A) Domain-level judgments for each individual RCT. (B) Summary of bias judgments across all domains and studies

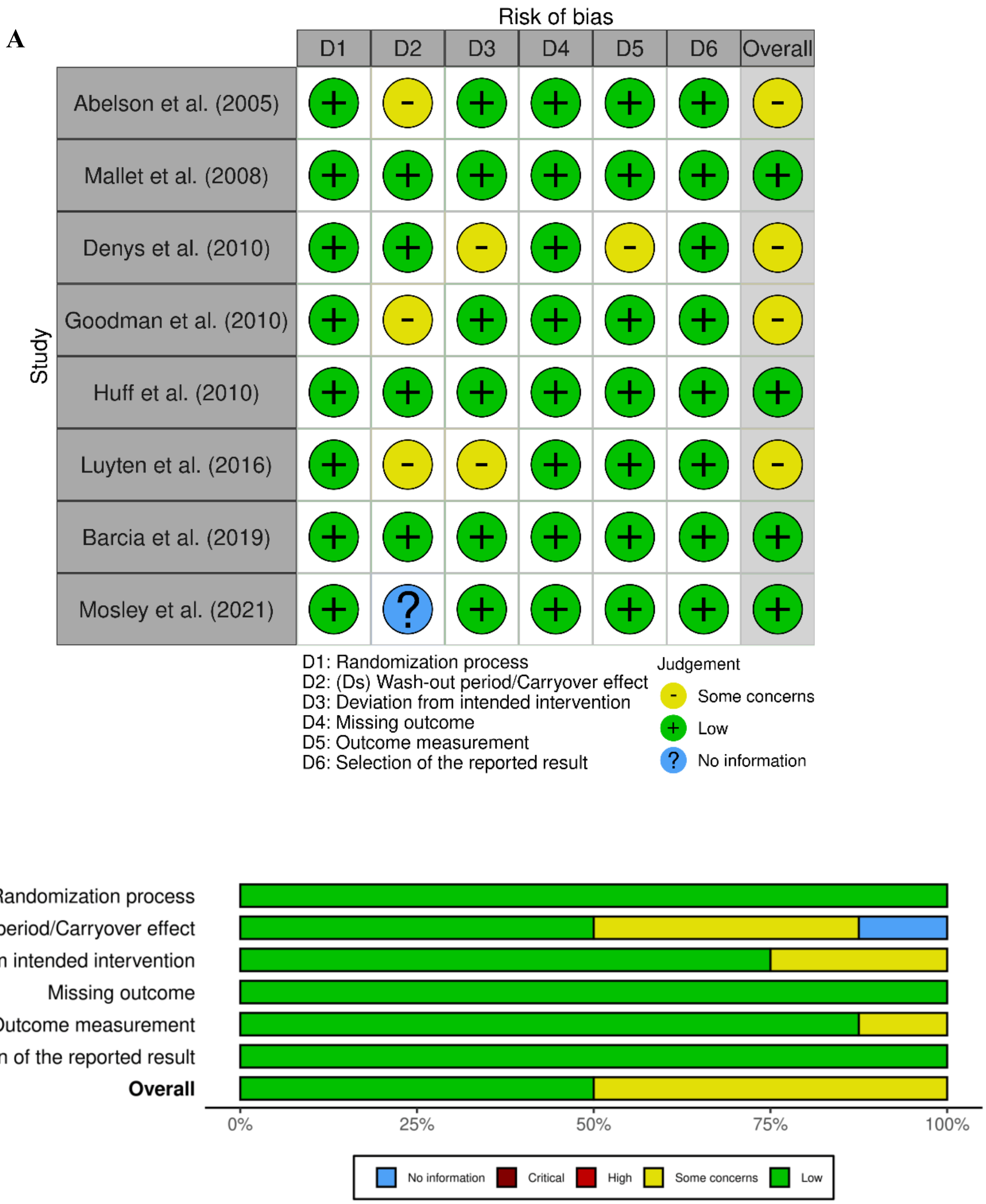

Figures were generated using the robvis visualization tool (McGuinness & Higgins, 2021).

Figure S2. Risk of bias assessment for non-randomized studies using the Cochrane ROBINS-I tool. (A) Domain-level judgments for each individual study. (B) Summary of bias judgments across all domains and studies.

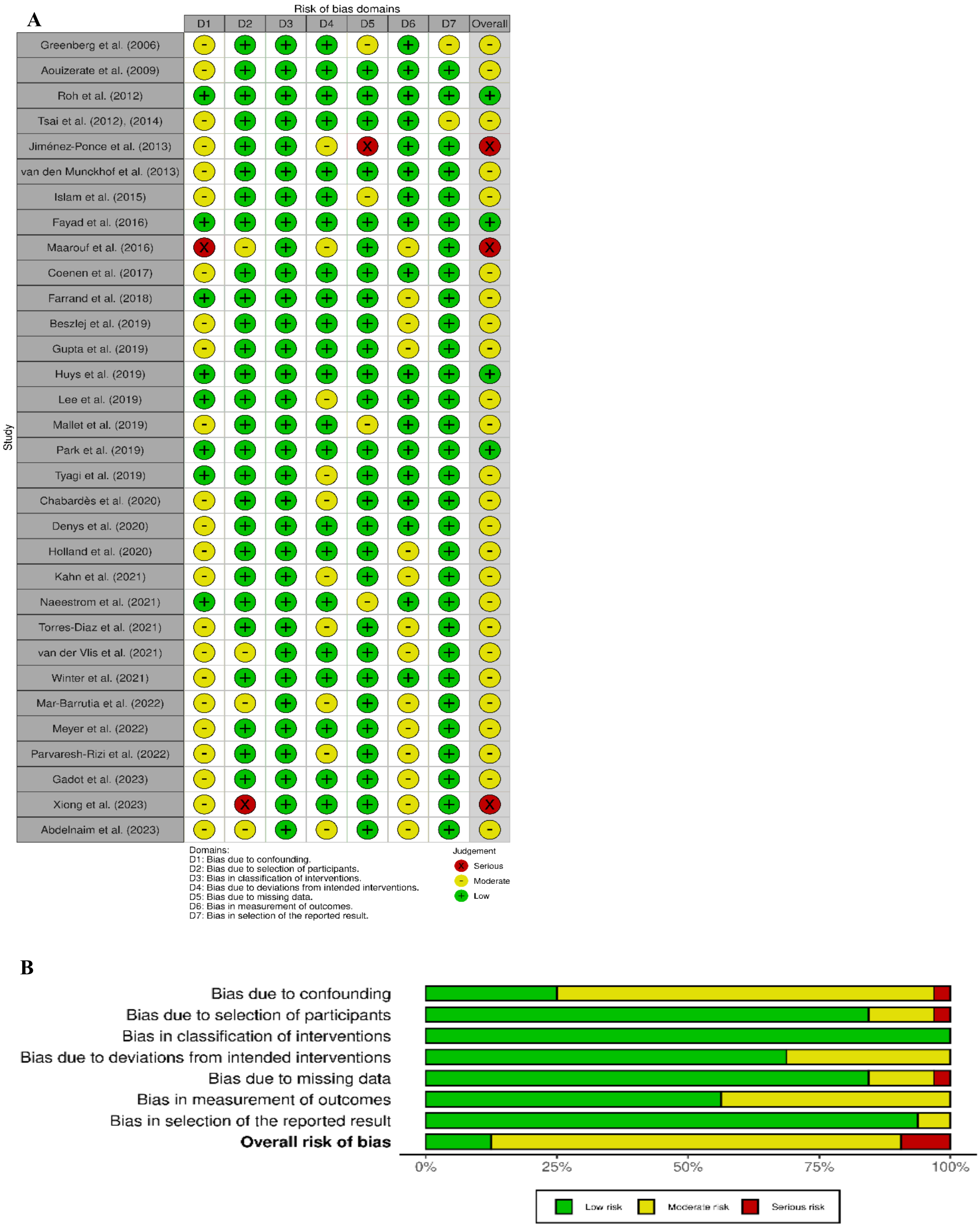

Figures were generated using the robvis visualization tool (McGuinness & Higgins, 2021).

Figure S3. Publication bias assessment for the mean difference in Y-BOCS score change in non-randomized studies at the last follow-up

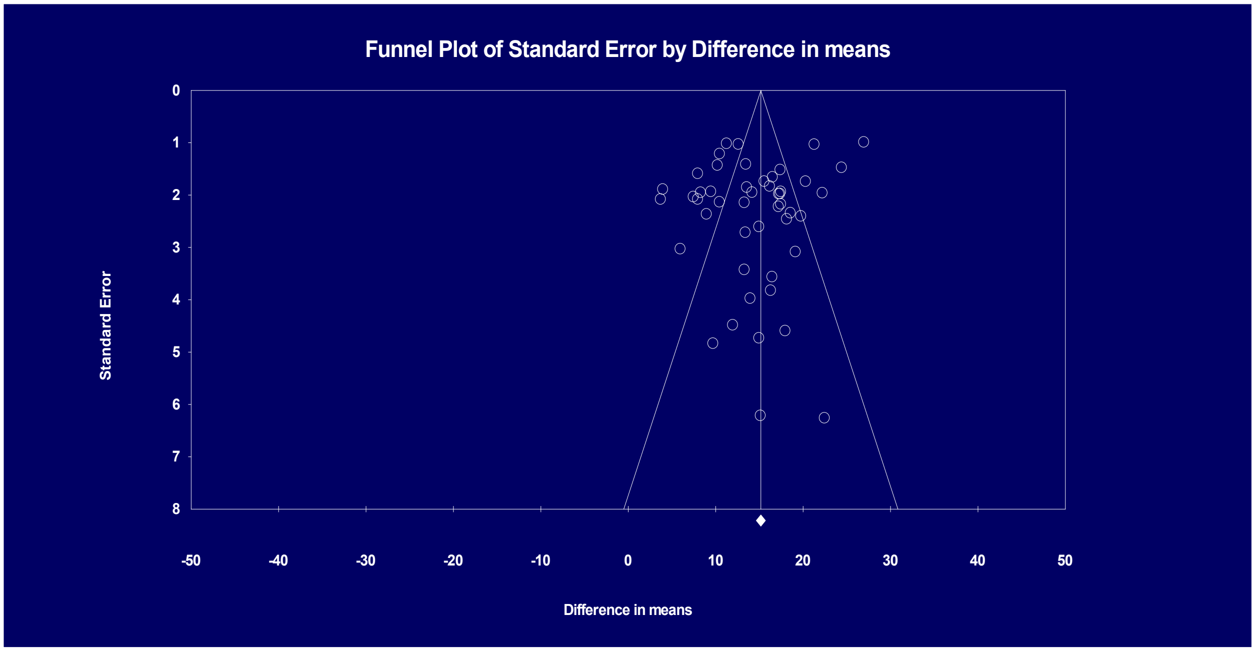

Figure S4. Meta-Regression of follow-up duration and mean difference in Y-BOCS scores in non-randomized studies at last follow-up

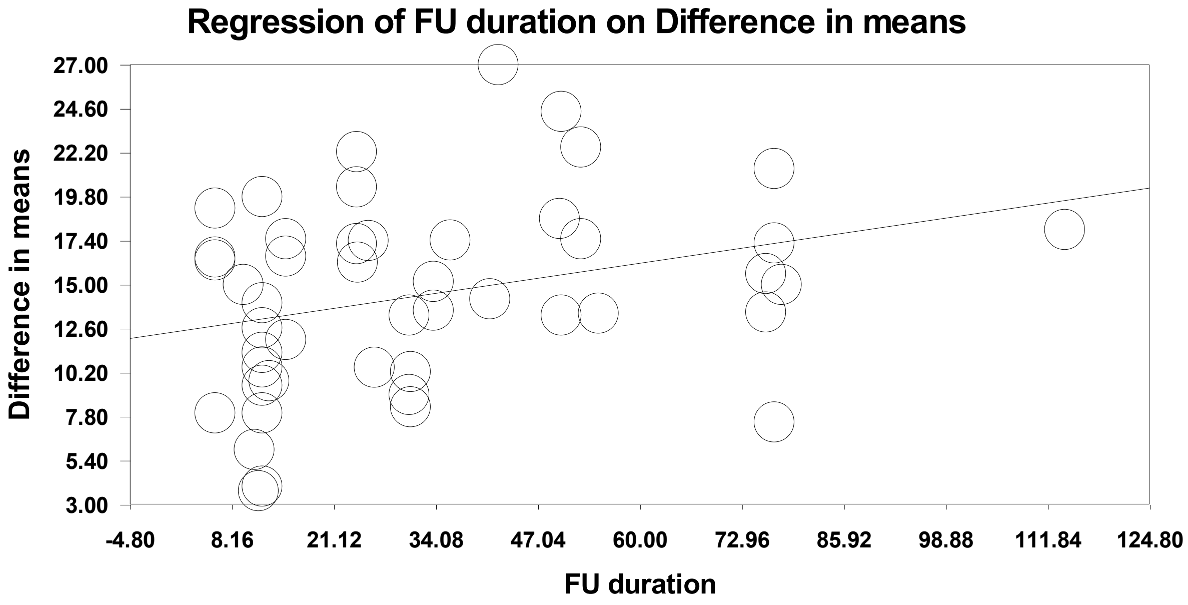

Quality of the evidence based on GRADE approach

Figure S5. GRADE assessment of the certainty of evidence from randomized controlled trials evaluating the efficacy of DBS based on the mean difference in Y-BOCS scores in TR-OCD

Outcome indicator	No. of Studies	Study Design	Risk of Bias[a]	Inconsistency	Indirectness	Imprecision	Publication Bias	Overall quality of evidence[b]	Effect (95% CI)
Y-BOCS reduction (mean difference)	8	RCT	Some concerns	Not serious	Not serious	Not serious	Undetected	Moderate ●●●○	MD = 6.9 points (95% CI: 4.5 to 9.4)

[a] Risk of bias assessment of individual RCTs, evaluated using the Cochrane RoB 2 tool across all domains.

[b] The GRADE system (Grading of Recommendations Assessment, Development and Evaluation) classifies the certainty of evidence into four levels based on the strength of the data supporting each outcome. High = It is very unlikely that further research will change the effect estimate. Moderate = Moderate confidence in the effect estimates; further research may change the estimate. Low = Limited confidence in the effect estimates; further research is likely to have an important impact. Very low = The true effect is likely to be substantially different from the estimate.

Abbreviations: Grade, Grading of Recommendations Assessment, Development and Evaluation; CI, confidence interval; Y-BOCS, Yale-Brown Obsessive Compulsive scale; RCT, randomized controlled trials; TR-OCD, treatment-resistant obsessive-compulsive disorder.

The certainty of evidence was downgraded by one level due to some concerns regarding risk of bias in the included RCTs. Most studies were rated as having some concerns related to methodology (wash-in-wash-out), blinding or randomization, but none were at high or critical risk. No additional downgrading was applied for inconsistency, indirectness, imprecision, or publication bias.

Risk of bias

Risk of bias was assessed individually for each randomized controlled trial comparing active DBS stimulation with sham or stimulation-OFF conditions, using the six domains of the Cochrane Risk of Bias 2.0 tool. This assessment considered the extent to which methodological limitations could affect the direction, magnitude, consistency, or accuracy of the reported outcomes—specifically, the reduction in OCD symptom severity as measured by the Y-BOCS. The majority of included RCTs were rated as having some concerns according to the risk of bias assessment. However, none were considered to be at high or critical risk.

The domain most frequently associated with concerns was related to the absence of washout periods and potential carryover effects. In one study[2], during the double-blind testing phase consisting of four consecutive 3-week ON or OFF stimulation periods without a washout between randomized periods. The sequence of stimulation and sham periods was not reported for two of the four patients (Patients 1 and 4). Moreover, the randomized allocation could have led to consecutive periods of active stimulation, thereby extending the total stimulation time compared to a single three-week period, which may have conferred a therapeutic advantage. In addition, the absence of a washout period between phases increases the risk of carryover effects, potentially leading to an overestimation or underestimation of the sham condition's effect. On the other hand, the relatively short duration of stimulation (three weeks) may have been insufficient to produce observable clinical effects in treatment-resistant patients, potentially explaining the lack of significant benefit. In another study (Goodman et al., 2010), the results observed during the three-month blinded phase in the group allocated to the ON–ON stimulation sequence (one month per phase) may have been influenced by the extended exposure to active stimulation, compared to the OFF–ON group, which received only one month of active stimulation. Indeed, patients S4 and S6, enrolled in the ON–ON group,

showed greater clinical improvement than those in the OFF–ON group. However, no statistically significant difference was found between the two groups. One additional RCT did not include a washout period or report an assessment of potential carryover effects (Luyten et al., 2016). Another RCT (Denys et al., 2010) also lacked a washout phase but reported no evidence of carryover effects. Concerns related to deviations from intended interventions were the second most frequent. In Denys et al. (2010), two participants did not complete the sham phase, and some outcomes were assessed by non-blinded raters. Observable stimulation effects may have compromised blinding in that study as well as in Luyten et al. (2016), where one participant withdrew early.

All other domains were judged to carry low risk of bias. However, due to methodological limitations potentially affecting the results, we downgraded the certainty of evidence by one level.

Inconsistency of results

High heterogeneity was observed across the randomized controlled trials ($I^2 = 62.93\%$), which likely reflects differences in study design and methodology, including variations in trial duration and the length of stimulation phases (ranging from two weeks to three months). Additional sources of heterogeneity may include differences in DBS optimization strategies and stimulation targets, such as the VC/VS, NAc, ALIC, or amSTN. GRADE guidance emphasizes that statistical heterogeneity alone should not be the decisive factor for downgrading the certainty of evidence, and that consistency should be assessed on the basis of the direction and magnitude of effects rather than the I^2 statistic alone (Guyatt et al., 2011). Despite this substantial statistical heterogeneity, the therapeutic effects of DBS consistently favored the active stimulation condition, although some did not achieve statistically significant symptom reduction. None of the included studies demonstrated a negative or worsening therapeutic effect of DBS. In contrast, no sham period demonstrated clinical improvements. Therefore, we did not downgrade the certainty of evidence for inconsistency.

Indirectness

All included RCTs enrolled adults with severe, TR-OCD (mean baseline Y-BOCS ≥ 31), closely corresponding to the target population of interest. The intervention (DBS) and comparator (sham stimulation or stimulation-off periods) were appropriate and aligned with the clinical question, which aimed to evaluate the effect of DBS on OCD symptom severity. All studies employed the Y-BOCS, a validated and clinically relevant outcome measure. Although the duration of the blinded stimulation phase varied across trials (ranging from 2 to 12 weeks), all consistently demonstrated greater symptom reduction during active DBS compared to sham condition. We judged that this variation does not compromise the applicability of the evidence. Therefore, we did not downgrade for indirectness.

Imprecision

Imprecision was not judged serious. The pooled mean difference was 6.9 points (95% CI: 4.5 to 9.4). Although the lower bound of the interval slightly overlaps the minimal clinically important difference for the Y-BOCS, which is typically estimated between 4 and 6 points in pharmacological studies (Cohen et al., 2025; Farris et al., 2013; Mataix-Cols et al., 2016), the effect size remained statistically significant and clinically meaningful. Therefore, the certainty of evidence was not downgraded for imprecision. All included studies showed a consistent direction of benefit, and no trial reported a worsening of symptoms under DBS. According to GRADE guidance, downgrading for imprecision is not warranted when the confidence interval excludes both the null effect and clinically unimportant effects, even in the context of small sample

sizes (Schünemann et al., 2013). Therefore, based on both statistical precision and clinical relevance, the certainty of the evidence was not downgraded for imprecision.

Publication bias

The presence of publication bias could not be ruled out due to the small number of studies (n = 8); however, no statistical or qualitative indication of bias was detected (Egger's test p = 0.133). We therefore judged the risk as undetected and did not downgrade the certainty of evidence.

Overall quality of evidence

The overall certainty of evidence was rated as moderate, downgraded by one level due to some concerns regarding risk of bias. Although imprecision was considered borderline given that the lower bound of the confidence interval (4.5 points) slightly overlaps the estimated minimal clinically important difference for the Y-BOCS (4 to 6 points), the effect size remained statistically and clinically meaningful. No additional downgrading was applied for inconsistency, indirectness, or publication bias.

Figure S6. GRADE assessment of the certainty of evidence from non-randomized studies evaluating the effectiveness of DBS based on the mean difference in Y-BOCS scores in TR-OCD

Outcome indicator	No. of Studies	Study Design	Risk of Bias[a]	Inconsistency	Indirectness	Imprecision	Publication Bias	Magnitude of effect	Overall quality of evidence[b]	Effect (95% CI)
Y-BOCS reduction (mean difference)	32	NON-RCT	Serious	Not serious	Not serious	Not serious	Undetected	Large	Low ●●○○	MD = 14.5 points (95% CI: 12.8 to 16.3)

[a] Risk of bias assessment of individual non-RCTs, evaluated using the Cochrane ROBIN-I tool across all domains.
[b] The GRADE system (Grading of Recommendations Assessment, Development and Evaluation) classifies the certainty of evidence into four levels based on the strength of the data supporting each outcome. High = It is very unlikely that further research will change the effect estimate. Moderate = Moderate confidence in the effect estimates; further research may change the estimate. Low = Limited confidence in the effect estimates; further research is likely to have an important impact. Very low = The true effect is likely to be substantially different from the estimate.

Abbreviations: Grade, Grading of Recommendations Assessment, Development and Evaluation; CI, confidence interval; Y-BOCS, Yale-Brown Obsessive Compulsive scale; non-RCT, non-randomized controlled trials; TR-OCD, treatment-resistant obsessive-compulsive disorder.

Risk of bias

Risk of bias was assessed individually for each non-randomized controlled trial or open-label phase following a randomized controlled phase, across the seven domains of the Cochrane ROBINS-I tool. This evaluation considered the extent to which methodological limitations could affect the direction, magnitude, consistency, or accuracy of the reported outcomes—specifically, the reduction in obsessive-compulsive symptom severity as measured by the Y-BOCS. Most of the included non-randomized studies were assessed as having low to moderate risk of bias, while three studies were rated as serious. Given their inclusion in the pooled effect and their potential influence on the magnitude of the observed effect, we conservatively downgraded the certainty of evidence by one level.

Inconsistency of results

Although substantial heterogeneity was observed across studies, with an I^2 of 89.79% overall and 80.4% when only studies with five or more participants were considered, reflecting considerable variability

in the magnitude of effect sizes, GRADE guidance emphasizes that statistical heterogeneity alone should not be the decisive factor for downgrading the certainty of evidence (Schünemann et al., 2022; Guyatt et al., 2011). Instead, the assessment of consistency should be based on visual inspection of the forest plot, statistical indicators, and consideration of factors contributing to heterogeneity. Despite the high I^2 value, the direction of effect consistently favored deep brain stimulation, with no studies reporting non-beneficial effects. The pooled mean difference was large and statistically precise (MD = 14.5 [95% CI: 12.8 to 16.3]), and the confidence interval was narrow. Furthermore, meta-regression analyses identified trial duration as a significant moderator, and subgroup analysis revealed significant differences in effect sizes across stimulation targets ($Q = 59.9$, $p = 0.0001$), both of which help explain the observed heterogeneity. Based on these considerations, and in line with a conservative approach, the certainty of evidence was not downgraded for inconsistency.

Indirectness

No concerns about indirectness. All included studies assessed the efficacy of DBS in TR-OCD using the Y-BOCS as the sole clinical outcome measure. Subgroup analyses based on stimulation target and sensitivity analyses including only studies with more than five participants were conducted to ensure clinical and anatomical consistency. These elements support the directness of the evidence, and therefore, we did not downgrade for indirectness.

Imprecision

No concerns were identified regarding imprecision. The pooled effect was statistically precise, with a mean difference of 14.5 Y-BOCS points [95% CI: 12.8 to 16.3], and a narrow confidence interval spanning only 3.5 points. Although no minimal important difference has been formally established for DBS in TR-OCD, previous pharmacological and behavioral trials have generally considered a 4 to 6 points reduction on the Y-BOCS scale as clinically meaningful (Cohen et al., 2025; Farris et al., 2013; Mataix-Cols et al., 2016). The observed MD of 14.5 points therefore indicates a substantial and clinically relevant treatment effect. At the last follow-up, data from 362 participants were included in the analysis (368 if counting participants stimulated at two different targets consecutively, as in Tyagi et al. (2019). While this number falls slightly below the GRADE-recommended threshold of 400 participants for optimal information size, it nonetheless approaches this benchmark. Given the magnitude and precision of the effect, the standardized use of the Y-BOCS across studies, and the narrow CI, we did not downgrade the certainty of evidence for imprecision.

Publication of bias

Publication bias was not detected. Visual inspection of the funnel plot did not reveal any notable asymmetry, and Egger's regression test, which was adequately powered given the number of studies, yielded a non-significant result ($p = 0.247$). Therefore, no downgrading was applied for publication bias.

Large effect

The pooled mean difference of 14.5 Y-BOCS points corresponds to a standardized mean difference (SMD) of 3.16, indicating a very large effect size. According to GRADE guidance, a very large effect observed in non-randomized studies may justify upgrading the certainty of evidence.

Overall

The certainty of evidence started at low due to the non-randomized study design. Adopting a conservative approach, one level was downgraded due to three studies being assessed at serious risk of bias. However, the evidence was upgraded by one level based on the very large observed effect size (MD = 14.5 Y-BOCS points [95% CI: 12.8 to 16.3]; SMD = 3.16), resulting in a final certainty rating of low.

Figure S7. DBS targets and stereotactic coordinates of stimulation sites based on anatomical reconstruction. A, axial plane; B, coronal plane. Red circles indicate the NAc (ventral striatum), green the putamen, orange the internal and external pallidum, dark green the amSTN, light blue, the caudate, cyan blue the thalamus, and dark red the red nucleus. Abbreviations: ALIC, anterior limb of the internal capsule; amSTN, antero-medial subthalamic nucleus; BNST, bed nucleus of the stria terminalis; ITP, inferior thalamic peduncle; slMFB, superolateral branch of the medial forebrain bundle; vALIC, ventral part of the internal capsule; VC/VS, ventral capsule/ventral striatum; NAc, nucleus accumbens.

A

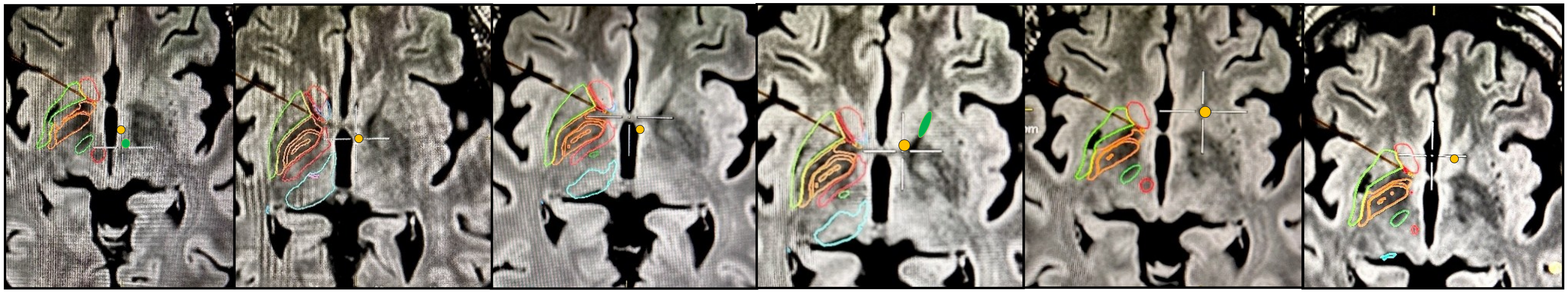

● slMFB: x =7.6*, y= -3*
● amSTN: x=11, y= 7/12 MCP

● ITP: x =6.5, y= -5

● BNST: x =3, y= -2

● Caudal VC/VS: x=8, y=0
● ALIC

● vALIC-NAc: x=13, y=7.5

● Rostral NAc: x=7, y=3

B

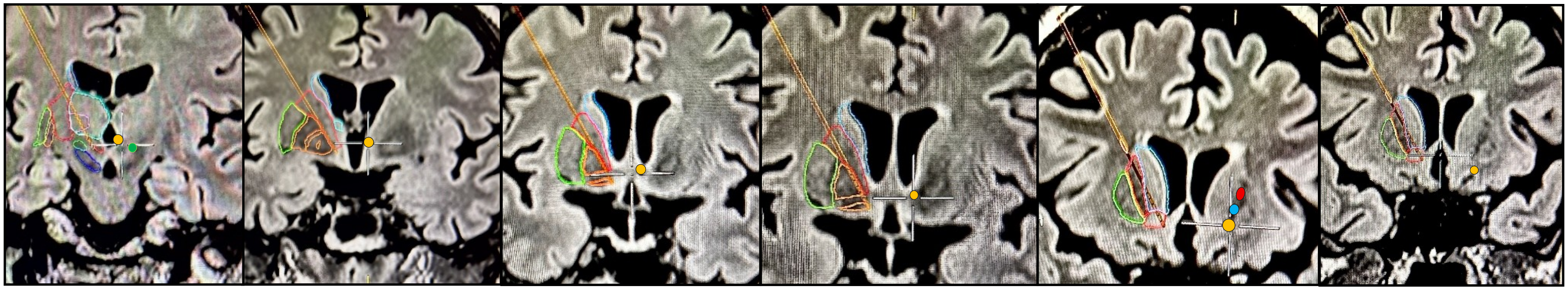

● slMFB: z= -3*
● amSTN: z= -2.2

● ITP: z= 0

● BNST: z= 0

● Caudal VC/VS: z= 0

● vALIC-NAc: z= -3
● vALIC: z= 3
● ALIC: z= 5

● Rostral NAc: z= -6

References

1. Abdelnaim, M.A.; Lang-Hambauer, V.; Hebel, T.; Schoisswohl, S.; Schecklmann, M.; Deuter, D.; Schlaier, J.; Langguth, B. Deep Brain Stimulation for Treatment Resistant Obsessive Compulsive Disorder; an Observational Study with Ten Patients under Real-Life Conditions. *Front. Psychiatry* **2023**, *14*, 1242566, doi:10.3389/fpsyt.2023.1242566.
2. Abelson, J.L.; Curtis, G.C.; Sagher, O.; Albucher, R.C.; Harrigan, M.; Taylor, S.F.; Martis, B.; Giordani, B. Deep Brain Stimulation for Refractory Obsessive-Compulsive Disorder. *Biological Psychiatry* **2005**, *57*, 510–516, doi:10.1016/j.biopsych.2004.11.042.
3. Aouizerate, B.; Cuny, E.; Bardinet, E.; Yelnik, J.; Martin-Guehl, C.; Rotge, J.-Y.; Rougier, A.; Bioulac, B.; Tignol, J.; Mallet, L.; et al. Distinct Striatal Targets in Treating Obsessive-Compulsive Disorder and Major Depression Case Report. *Journal of Neurosurgery* **2009**, *111*, 775–779, doi:10.3171/2009.2.JNS0881.
4. Avery, S.; Clauss, J.; Blackford, J. The Human BNST: Functional Role in Anxiety and Addiction. *Neuropsychopharmacology* **2016**, *41*, 126–141.
5. Barcia, J.A.; Avecillas-Chasin, J.M.; Nombela, C.; Arza, R.; Garcia-Albea, J.; Pineda-Pardo, J.A.; Reneses, B.; Strange, B.A. Personalized Striatal Targets for Deep Brain Stimulation in Obsessive-Compulsive Disorder. *Brain Stimulation* **2019**, *12*, 724–734, doi:10.1016/j.brs.2018.12.226.
6. Beszlej, J.A.; Siwicki, D.; Fila-Witecka, K.; Wieczorek, T.; Piotrowski, P.; Weiser, A.; Tabakow, P.; Rymaszewska, J. Deep Brain Stimulation in Obsessive-Compulsive Disorder - Case Report of Two Patients. *Psychiatria Polska* **2019**, *53*, 807–824, doi:10.12740/PP/104643.
7. Chabardes, S.; Krack, P.; Piallat, B.; Bougerol, T.; Seigneuret, E.; Yelnik, J.; al Deep Brain Stimulation of the Subthalamic Nucleus in Obsessive–Compulsive Disorders: Long-Term Follow-up of an Open, Prospective, Observational Cohort. *J Neurol Neurosurg Psychiatry* **2020**, *91*, 1349–1356.
8. Coenen, V.A.; Döbrössy, M.D.; Teo, S.J.; Wessolleck, J.; Sajonz, B.E.A.; Reinacher, P.C.; Thierauf-Emberger, A.; Spittau, B.; Leupold, J.; Von Elverfeldt, D.; et al. Diverging Prefrontal Cortex Fiber Connection Routes to the Subthalamic Nucleus and the Mesencephalic Ventral Tegmentum Investigated with Long Range (Normative) and Short Range (Ex-Vivo High Resolution) 7T DTI. *Brain Struct Funct* **2022**, *227*, 23–47, doi:10.1007/s00429-021-02373-x.
9. Coenen, V.A.; Schlaepfer, T.E.; Goll, P.; Reinacher, P.C.; Voderholzer, U.; Tebartz Van Elst, L.; Urbach, H.; Freyer, T. The Medial Forebrain Bundle as a Target for Deep Brain Stimulation for Obsessive-Compulsive Disorder. *CNS Spectr.* **2017**, *22*, 282–289, doi:10.1017/S1092852916000286.
10. Cohen, S.; Niemeijer, M.; Zantvoord, J.; van Wingen, G.; Mocking, R.; Denys, D. Deep Brain Stimulation for Obsessive-Compulsive-Disorder: A Systematic Review and Meta-Analysis of Individual Participant Outcome Data from Sham-Controlled Trials. *Molecular Psychiatry* **2025**, *30*, 4937–4947, doi:10.1038/s41380-025-03092-z.
11. Denys, D.; Graat, I.; Mocking, R.; De Koning, P.; Vulink, N.; Figee, M.; Ooms, P.; Mantione, M.; Van Den Munckhof, P.; Schuurman, R. Efficacy of Deep Brain Stimulation of the Ventral Anterior Limb of the Internal Capsule for Refractory Obsessive-Compulsive Disorder: A Clinical Cohort of 70 Patients. *American Journal of Psychiatry* **2020**, *177*, 265 EP – 271, doi:10.1176/appi.ajp.2019.19060656.
12. Denys, D.; Mantione, M.; Figee, M.; Van Den Munckhof, P.; Koerselman, F.; Westenberg, H.; Bosch, A.; Schuurman, R. Deep Brain Stimulation of the Nucleus Accumbens for Treatment-Refractory Obsessive-Compulsive Disorder. *Arch Gen Psychiatry* **2010**, *67*, 1061, doi:10.1001/archgenpsychiatry.2010.122.
13. Farrand, S.; Evans, A.H.; Mangelsdorf, S.; Loi, S.M.; Mocellin, R.; Borham, A.; Bevilacqua, J.; Blair-West, S.; Walterfang, M.A.; Bittar, R.G.; et al. Deep Brain Stimulation for Severe Treatment-Resistant Obsessive-Compulsive Disorder: An Open-Label Case Series. *Australian and New Zealand Journal of Psychiatry* **2018**, *52*, 699–708, doi:10.1177/0004867417731819.
14. Farris, S.; McLean, C.; Van Meter, P.; Simpson, H.B.; Foa, E. Treatment Response, Symptom Remission, and Well-

ness in Obsessive-Compulsive Disorder. *The Journal of Clinical Psychiatry* **2013**, *74*, 685–690.
15. Fayad, S.M.; Guzick, A.G.; Reid, A.M.; Mason, D.M.; Bertone, A.; Foote, K.D.; Okun, M.S.; Goodman, W.K.; Ward, H.E. Six-Nine Year Follow-up of Deep Brain Stimulation for Obsessive-Compulsive Disorder. *PLoS ONE* **2016**, *11*, e0167875, doi:10.1371/journal.pone.0167875.
16. Gadot, R.; Li, N.; Shofty, B.; Avendano-Ortega, M.; McKay, S.; Bijanki, K.R.; Robinson, M.E.; Banks, G.; Provenza, N.; Storch, E.A.; et al. Tractography-Based Modeling Explains Treatment Outcomes in Patients Undergoing Deep Brain Stimulation for Obsessive-Compulsive Disorder. *Biological Psychiatry* **2024**, *96*, 95–100, doi:10.1016/j.biopsych.2023.01.017.
17. Goodman, W.K.; Foote, K.D.; Greenberg, B.D.; Ricciuti, N.; Bauer, R.; Ward, H.; Shapira, N.A.; Wu, S.S.; Hill, C.L.; Rasmussen, S.A.; et al. Deep Brain Stimulation for Intractable Obsessive Compulsive Disorder: Pilot Study Using a Blinded, Staggered-Onset Design. *Biological Psychiatry* **2010**, *67*, 535–542, doi:10.1016/j.biopsych.2009.11.028.
18. Graat, I.; Mocking, R.; Figee, M.; Vulink, N.; de Koning, P.; Ooms, P.; Mantione, M.; van den Munckhof, P.; Schuurman, R.; Denys, D. Long-Term Outcome of Deep Brain Stimulation of the Ventral Part of the Anterior Limb of the Internal Capsule in a Cohort of 50 Patients With Treatment-Refractory Obsessive-Compulsive Disorder. *Biological Psychiatry* **2021**, *90*, 714–720, doi:10.1016/j.biopsych.2020.08.018.
19. Greenberg, B.; Malone, D.; Friehs, G.; Rezai, A.; Kubu, C.; Malloy, P.; al Three-Year Outcomes in Deep Brain Stimulation for Highly Resistant Obsessive–Compulsive Disorder. *Neuropsychopharmacology* **2006**, *31*, 2384–2393.
20. Gupta, A.; Khanna, S.; Jain, R. Deep Brain Stimulation of Ventral Internal Capsule for Refractory Obsessive–Compulsive Disorder. *Indian J Psychiatry* **2019**, *61*, 532–536.
21. Holland, M.T.; Trapp, N.T.; McCormick, L.M.; Jareczek, F.J.; Zanaty, M.; Close, L.N.; Beeghly, J.; Greenlee, J.D.W. Deep Brain Stimulation for Obsessive-Compulsive Disorder : A Long Term Naturalistic Follow Up Study in a Single Institution. *Frontiers in Psychiatry* **2020**, *11*, doi:10.3389/fpsyt.2020.00055.
22. Huff, W.; Lenartz, D.; Schormann, M.; Lee, S.H.; Kuhn, J.; Koulousakis, A.; Mai, J.; Daumann, J.; Maarouf, M.; Klosterkotter, J.; et al. Unilateral Deep Brain Stimulation of the Nucleus Accumbens in Patients with Treatment-Resistant Obsessive-Compulsive Disorder: Outcomes after One Year. *Clinical Neurology and Neurosurgery* **2010**, *112*, 137–143, doi:10.1016/j.clineuro.2009.11.006.
23. Huys, D.; Kohl, S.; Baldermann, J.C.; Timmermann, L.; Sturm, V.; Visser-Vandewalle, V.; Kuhn, J. Open-Label Trial of Anterior Limb of Internal Capsule-Nucleus Accumbens Deep Brain Stimulation for Obsessive-Compulsive Disorder : Insights Gained. *Journal of Neurology, Neurosurgery and Psychiatry* **2019**, *90*, 805–812, doi:10.1136/jnnp-2018-318996.
24. Islam, L.; Franzini, A.; Messina, G.; Scarone, S.; Gambini, O. Deep Brain Stimulation of the Nucleus Accumbens and Bed Nucleus of Stria Terminalis for Obsessive-Compulsive Disorder: A Case Series. *World Neurosurgery* **2015**, *83*, 657–663, doi:10.1016/j.wneu.2014.12.024.
25. Jimenez, F.; Nicolini, H.; Lozano, A.M.; Piedimonte, F.; Salin, R.; Velasco, F. Electrical Stimulation of the Inferior Thalamic Peduncle in the Treatment of Major Depression and Obsessive Compulsive Disorders. *World Neurosurgery* **2013**, *80*, S30.e17-S30.e25, doi:10.1016/j.wneu.2012.07.010.
26. Kahn, L.; Sutton, B.; Winston, H.R.; Abosch, A.; Thompson, J.A.; Davis, R.A. Deep Brain Stimulation for Obsessive-Compulsive Disorder: Real World Experience Post-FDA-Humanitarian Use Device Approval. *Frontiers in Psychiatry* **2021**, *12*, 568932, doi:10.3389/fpsyt.2021.568932.
27. Lee, D.J.; Dallapiazza, R.F.; De Vloo, P.; Elias, G.J.B.; Fomenko, A.; Boutet, A.; Giacobbe, P.; Lozano, A.M. Inferior Thalamic Peduncle Deep Brain Stimulation for Treatment-Refractory Obsessive-Compulsive Disorder : A Phase 1 Pilot Trial. *Brain Stimulation* **2019**, *12*, 344–352, doi:10.1016/j.brs.2018.11.012.
28. Lucas-Neto, L.; Neto, D.; Oliveira, E.; Martins, H.; Mourato, B.; Correia, F.; Rainha-Campos, A.; Goncalves-Ferreira, A. Three Dimensional Anatomy of the Human Nucleus Accumbens. *Acta Neurochirurgica* **2013**, *155*, 2389–2398, doi:10.1007/s00701-013-1820-z.

29. Luyten, L.; Hendrickx, S.; Raymaekers, S.; Gabriels, L.; Nuttin, B. Electrical Stimulation in the Bed Nucleus of the Stria Terminalis Alleviates Severe Obsessive-Compulsive Disorder. *Molecular Psychiatry* **2016**, *21*, 1272–1280, doi:10.1038/mp.2015.124.
30. Maarouf, M.; Neudorfer, C.; El Majdoub, F.; Lenartz, D.; Kuhn, J.; Sturm, V. Deep Brain Stimulation of Medial Dorsal and Ventral Anterior Nucleus of the Thalamus in OCD: A Retrospective Case Series. *PLoS ONE* **2016**, *11*, e0160750, doi:10.1371/journal.pone.0160750.
31. Mai, J.K.; Assheuer, J.; Paxinos, G. *Atlas of the Human Brain*; 2nd ed.; Elsevier Academic Press: Amsterdam ; Boston, 2004; ISBN 978-0-12-465741-0.
32. Mai, J.K.; Majtanik, M.; Paxinos, G. *Atlas of the Human Brain*; 4th ed.; Elsevier Science: s.l, 2015; ISBN 978-0-12-802801-8.
33. Mallet, L.; Du Montcel, S.T.; Clair, A.-H.; Arbus, C.; Bardinet, E.; Baup, N.; Chabardes, S.; Chereau, I.; Czernecki, V.; Fontaine, D.; et al. Long-Term Effects of Subthalamic Stimulation in Obsessive-Compulsive Disorder: Follow-up of a Randomized Controlled Trial. *Brain Stimulation* **2019**, *12*, 1080–1082, doi:10.1016/j.brs.2019.04.004.
34. Mallet, L.; Polosan, M.; Jaafari, N.; Baup, N.; Welter, M.-L.; Fontaine, D.; du Montcel, S.T.; Yelnik, J.; Chereau, I.; Arbus, C.; et al. Subthalamic Nucleus Stimulation in Severe Obsessive-Compulsive Disorder. *New England Journal of Medicine* **2008**, *359*, 2121–2134, doi:10.1056/NEJMoa0708514.
35. Mar-Barrutia, L.; Ibarrondo, O.; Mar, J.; Real, E.; Segalàs, C.; Bertolín, S.; Aparicio, M.A.; Plans, G.; Menchón, J.M.; Alonso, P. Long-Term Comparative Effectiveness of Deep Brain Stimulation in Severe Obsessive-Compulsive Disorder. *Brain Stimul* **2022**, *15*, 1128–1138, doi:10.1016/j.brs.2022.07.050.
36. Mataix-Cols, D.; Fernández de la Cruz, L.; Nordsletten, A.E.; Lenhard, F.; Kayoko, I.; Simpson, H.B. Towards an International Expert Consensus for Defining Treatment Response, Remission, Recovery and Relapse in Obsessive-Compulsive Disorder. *World Psychiatry* **2016**, *15*, 80–81, doi:10.1002/wps.20299.
37. Mavridis, I.N. Nucleus Accumbens Stereotaxy for Deep Brain Stimulation: Anatomical Focus. *Acta neurochirurgica* **2014**, *156*, 589–591, doi:10.1007/s00701-013-1883-x.
38. McGuinness, L.A.; Higgins, J.P.T. Risk-of-bias VISualization (Robvis): An R Package and Shiny Web App for Visualizing Risk-of-bias Assessments. *Research Synthesis Methods* **2021**, *12*, 55–61, doi:10.1002/jrsm.1411.
39. Menchón, J.M.; Real, E.; Alonso, P.; Aparicio, M.A.; Segalas, C.; Plans, G.; Luyten, L.; Brunfaut, E.; Matthijs, L.; Raymakers, S.; et al. A Prospective International Multi-Center Study on Safety and Efficacy of Deep Brain Stimulation for Resistant Obsessive-Compulsive Disorder. *Mol Psychiatry* **2021**, *26*, 1234–1247, doi:10.1038/s41380-019-0562-6.
40. Meyer, D.M.; Spanier, S.; Kilian, H.M.; Reisert, M.; Urbach, H.; Sajonz, B.Ea.; Reinacher, P.C.; Normann, C.; Domschke, K.; Coenen, V.A.; et al. Efficacy of Superolateral Medial Forebrain Bundle Deep Brain Stimulation in Obsessive-Compulsive Disorder. *Brain Stimulation* **2022**, *15*, 582–585, doi:10.1016/j.brs.2022.03.004.
41. Morel, A. *Stereotactic Atlas of the Human Thalamus and Basal Ganglia*; 0 ed.; CRC Press, 2007; ISBN 978-0-429-19116-9.
42. Mosley, P.E.; Windels, F.; Morris, J.; Coyne, T.; Marsh, R.; Giorni, A.; Mohan, A.; Sachdev, P.; O'Leary, E.; Boschen, M.; et al. A Randomised, Double-Blind, Sham-Controlled Trial of Deep Brain Stimulation of the Bed Nucleus of the Stria Terminalis for Treatment-Resistant Obsessive-Compulsive Disorder. *Translational Psychiatry* **2021**, *11*, 190, doi:10.1038/s41398-021-01307-9.
43. Naesström, M.; Hariz, M.; Strömsten, L.; Bodlund, O.; Blomstedt, P. Deep Brain Stimulation in the Bed Nucleus of Stria Terminalis in Obsessive-Compulsive Disorder—1-Year Follow-Up. *World Neurosurgery* **2021**, *149*, e794–e802, doi:10.1016/j.wneu.2021.01.097.
44. Neto, L.L.; Oliveira, E.; Correia, F.; Ferreira, A.G. The Human Nucleus Accumbens: Where Is It? A Stereotactic, Anatomical and Magnetic Resonance Imaging Study. *Neuromodulation: Technology at the Neural Interface* **2008**, *11*, 13–22, doi:10.1111/j.1525-1403.2007.00138.x.
45. Park, H.R.; Kim, I.H.; Kang, H.; McCairn, K.W.; Lee, D.S.; Kim, B.-N.; Kim, D.G.; Paek, S.H. Electrophysiological

and Imaging Evidence of Sustained Inhibition in Limbic and Frontal Networks Following Deep Brain Stimulation for Treatment Refractory Obsessive Compulsive Disorder. *PLoS ONE* **2019**, *14*, e0219578, doi:10.1371/journal.pone.0219578.

46. Parvaresh- Rizi, M.; Ghadirivasfi, M.; Babaei, S.; Bagher Saberi Zafarghandi, M.; Fattahi, A.; Amirhasan Habibi, S.; Rohani, M.; Arezoomandan, R. Psychopathological and Neuropsychological Outcomes of Deep Brain Stimulation for Severe- Treatment-Resistant Obsessive-Compulsive Disorder: An Open-Label Case Series. *Journal of Clinical Neuroscience* **2022**, *98*, 229–234, doi:10.1016/j.jocn.2022.02.015.
47. Roh, D.; Chang, W.S.; Chang, J.W.; Kim, C.-H. Long-Term Follow-up of Deep Brain Stimulation for Refractory Obsessive-Compulsive Disorder. *Psychiatry Research* **2012**, *200*, 1067–1070, doi:10.1016/j.psychres.2012.06.018.
48. Schünemann, H.; Oxman, A.; Brożek, J.; Glasziou, P.; Jaeschke, R.; Vist, G.; Williams, J.; Craig, J.; Montori, V.; Bossuyt, P.; et al. *GRADE Handbook for Grading Quality of Evidence and Strength of Recommendations*; The GRADE Working Group; 2013;
49. Schünemann, H.J.; Neumann, I.; Hultcrantz, M.; Brignardello-Petersen, R.; Zeng, L.; Murad, M.H.; Izcovich, A.; Morgano, G.P.; Baldeh, T.; Santesso, N.; et al. GRADE Guidance 35: Update on Rating Imprecision for Assessing Contextualized Certainty of Evidence and Making Decisions. *Journal of Clinical Epidemiology* **2022**, *150*, 225–242, doi:10.1016/j.jclinepi.2022.07.015.
50. Torres Díaz, C.V.; Treu, S.; Strange, B.; Lara, M.; Navas, M.; Ezquiaga, E.; Zazo, E.S.; Vicente, J.S.; Muñiz, I.; Fernandez, F.S. Deep Brain Stimulation of the Nucleus Accumbens, Ventral Striatum, or Internal Capsule Targets for Medication-Resistant Obsessive-Compulsive Disorder: A Multicenter Study. *World Neurosurgery* **2021**, *155*, e168–e176, doi:10.1016/j.wneu.2021.08.039.
51. Tyagi, H.; Apergis-Schoute, A.M.; Akram, H.; Foltynie, T.; Limousin, P.; Drummond, L.M.; Fineberg, N.A.; Matthews, K.; Jahanshahi, M.; Robbins, T.W.; et al. A Randomized Trial Directly Comparing Ventral Capsule and Anteromedial Subthalamic Nucleus Stimulation in Obsessive-Compulsive Disorder: Clinical and Imaging Evidence for Dissociable Effects. *Biological Psychiatry* **2019**, *85*, 726–734, doi:10.1016/j.biopsych.2019.01.017.
52. van den Munckhof, P.; Bosch, D.; Mantione, M.; Figee, M.; Denys, D.; Schuurman, P. Active Stimulation Site of Nucleus Accumbens Deep Brain Stimulation in Obsessive–Compulsive Disorder Is Localized in the Ventral Internal Capsule. In *Stereotactic and Functional Neurosurgery*; Nikkhah, G., Pinsker, M. (eds), Eds.; Acta Neurochirurgica Supplement; Springer Vienna: Vienna, 2013; Vol. 117, pp. 53–59 ISBN 978-3-7091-1481-0.
53. Van Der Vlis, T.A.M.B.; Ackermans, L.; Mulders, A.E.P.; Vrij, C.A.; Schruers, K.; Temel, Y.; Duits, A.; Leentjens, A.F.G. Ventral Capsule/Ventral Striatum Stimulation in Obsessive-Compulsive Disorder: Toward a Unified Connectomic Target for Deep Brain Stimulation? *Neuromodulation: Technology at the Neural Interface* **2021**, *24*, 316–323, doi:10.1111/ner.13339.
54. Welter, M.-L.; Alves Dos Santos, J.F.; Clair, A.-H.; Lau, B.; Diallo, H.M.; Fernandez-Vidal, S.; Belaid, H.; Pelissolo, A.; Domenech, P.; Karachi, C.; et al. Deep Brain Stimulation of the Subthalamic, Accumbens, or Caudate Nuclei for Patients With Severe Obsessive-Compulsive Disorder: A Randomized Crossover Controlled Study. *Biological Psychiatry* **2021**, *90*, e45–e47, doi:10.1016/j.biopsych.2020.07.013.
55. Winter, L.; Saryyeva, A.; Schwabe, K.; Heissler, H.E.; Runge, J.; Alam, M.; Heitland, I.; Kahl, K.G.; Krauss, J.K. Long-Term Deep Brain Stimulation in Treatment-Resistant Obsessive-Compulsive Disorder: Outcome and Quality of Life at Four to Eight Years Follow-Up. *Neuromodulation: Technology at the Neural Interface* **2021**, *24*, 324–330, doi:10.1111/ner.13232.
56. Xiong, B.; Li, B.; Wen, R.; Gao, Y.; Gong, F.; Li, D.; Xu, Y.; Deng, H.; Xiao, L.; Yin, S.; et al. Use of Differential Stimulation of the Nucleus Accumbens and Anterior Limb of the Internal Capsule to Improve Outcomes of Obsessive-Compulsive Disorder. *J Neurosurg* **2023**, *139*, 1376–1385, doi:10.3171/2023.4.JNS221824%20Abstract.
